# Fresh *Phyllanthus emblica* (Amla) Fruit Supplementation Enhances Milk Fatty Acid Profiles and the Antioxidant Capacities of Milk and Blood in Dairy Cows

**DOI:** 10.3390/antiox11030485

**Published:** 2022-02-28

**Authors:** Mekonnen Tilahun, Liansheng Zhao, Lingling Sun, Yifan Shen, Lu Ma, Todd R. Callaway, Jianchu Xu, Dengpan Bu

**Affiliations:** 1 State Key Laboratory of Animal Nutrition, Institute of Animal Science, Chinese Academy of Agricultural Sciences, Beijing 100193, China; dmtilahun84@gmail.com (M.T.); zhaoliansheng@caas.cn (L.Z.); sunlingling114289@163.com (L.S.); shenswjs@126.com (Y.S.); malu@caas.cn (L.M.); jxu@mail.kib.ac.cn (J.X.); 2 Key Laboratory of Economic Plants and Biotechnology, Kunming Institute of Botany, Chinese Academy of Sciences, Kunming 650201, China; 3 Department of Animal and Dairy Science, University of Georgia, Athens, GA 30602-2771, USA; Todd.Callaway@uga.edu; 4 World Agroforestry Centre East and Central Asia, Kunming 650201, China; 5 Joint Laboratory on Integrated Crop-Tree-Livestock Systems of the Chinese Academy of Agricultural Sciences (CAAS), Ethiopian Institute of Agricultural Research (EIAR) and World Agroforestry Center (ICRAF), Beijing 100193, China

**Keywords:** amla, atherogenicity index, biohydrogenation, metabolites, polyphenols, UPLC-ESI-MS/MS

## Abstract

The objective of this study was to investigate the effect of a diet supplemented with fresh amla fruit as a natural feed additive on blood metabolic parameters, milk antioxidant capacity, and milk fatty acid (FA) proportions in lactating dairy cows. Eight ruminally cannulated mid-lactation dairy cows were used in a repeated crossover design. The first group of four cows received total mixed ration (TMR) feed without fresh amla fruit (control group). The remaining four cows sequentially supplemented fresh amla fruit (FAF) at three levels (200, 400, then 600 g/d) (treatment group) at 14-day intervals. In second period, control and treatment groups were exchanged. The first ten days were adjusted to diet adaptation for each sub-period, and the last four days for sampling milk and blood. A total of 514 metabolites were detected from FAF using UPLC-ESI-MS/MS. The five main metabolites in FAF were phenolic acids (22%), flavonoids (20%), lipids (20%), amino acids and derivatives (9%), and tannins (7%). Amla fruit supplementation reduced total saturated fatty acid and the omega-6/omega-3 ratio at 200 or 400 g/d FAF dose compared to controls. In addition, amla fruit increased unsaturated FA, such as C_20:5_ (Eicosapentaenoic acid, EPA) and C_22:6_ (docosahexaenoic acid, DHA), and branched-chain FA in a dose-dependent manner at 200 or 400 g/d compared to controls. In addition, amla fruit increased the antioxidant capacity biomarkers in the blood, such as superoxide dismutase (SOD) and albumin; this confirms that amla fruit is an excellent antioxidant, inhibiting reactive oxygen species’ (ROS) metabolism, and can thereby protect cells from oxidative stress. Moreover, the most remarkable improvement of ferric reducing-antioxidant power (FRAP) and total antioxidant capacity (TAC) in milk was recorded at 400 g/d FAF doses compared to controls. Therefore, fresh amla fruit doses for lactating cows at 400 g/d on an as-fed basis can be used as an alternative additive feed in dairy cow diets to improve antioxidant capacity, protein efficiency, butter quality, and to produce more desirable milk fatty acid profiles for human consumption.

## 1. Introduction

Plants and fruits naturally contain polyphenols such as tannins, flavonoids, and other phytochemicals that have antioxidant, anti-inflammatory, antibacterial, antiviral, and anti-parasitic properties that can boost the health and productivity of animals [1,2,3]. In addition, mixing phytogenics or tannins in ruminant animals’ diets has been found to modulate ruminal biohydrogenation, resulting in increased levels of health-promoting fatty acids in milk [4,5,6,7,8], such as very long chain n-3 fatty acids (FA) including eicosapentaenoic acid (EPA; 20:5n-3) and docosahexaenoic acid (DHA; C_22:6_). Moreover, tannins, when administered at low concentrations, can increase the production of microbially generated odd and branched FAs (OBCFA) (iso-and ante-iso and uneven chain FA) [9]. Milk OBCFAs are beneficial for human patients with cardiovascular diseases and type II diabetes [10]. Moreover, by using a variety of health index values, including atherogenicity index (AI) and hypocholesterolemic and hypercholesterolemic ratios (h/H), we can understand that milk fats with low AI values and high h/H have a lower cardiovascular disease risk [11,12].

Furthermore, natural phenolic compounds can be transferred from plant diets to ruminant milk, increasing milk antioxidant capacity and providing a promising strategy for improving product quality [13,14,15]. Antioxidant capacity is essential for decreasing risks of various diseases and mortality [16,17]. Therefore, polyphenol supplementation can produce a beneficial effect by making milk products healthier for human consumption. However, specific studies for each plant must be made, and caution should be applied to including polyphenols in ruminant diets since they may influence ruminants’ food intake, metabolic parameters, and hormonal regulation [18].

*Emblica officinalis* Gaertn. syn. *Phyllanthus emblica* L., universally known as ‘Amla’ or ‘Aonla’ or ‘Indian gooseberry’, is a popular fruit tree belonging to the family *Euphorbiaceae* and order *Geraniales* [19]. Amla originates from India but is also cultivated in several other tropical and sub-tropical countries such as Bangladesh, China (southern part), Malaysia, Mascarene Islands, Myanmar, Pakistan, Sri Lanka, and Uzbekistan [20]. Polyphenols, tannins, flavonoids, and ascorbic acid are amla’s primary antioxidants and bioactive components [19,21,22]. The tannin content of amla is 4% of fresh fruit [23], although this figure can be up to 35% in dried fruit powder [24], and hydrolysable tannins dominate amla fruits’ phenolic compounds [25,26,27].

We previously reported that fresh amla fruit supplementation did not affect feed intake by Holstein dairy cows, but it had a dose-dependent effect on milk production, milk composition, apparent nutrient digestibility, milk nitrogen efficiency, milk urea nitrogen, ruminal short chain fatty acid production, ammonia production, and protozoal counts [28]. In a study performed on buffalo calves, phytogenic additives containing amla fruit enhanced immune status, and increased metabolite concentrations in the blood, such as total protein, albumin, globulin, aspartate transaminase, and alanine transaminase [29,30]. According to Lozano-Sánchez et al. [31], the herbal additive that contains amla fruits does not affect metabolites (glucose, urea, cholesterol) related to energy or protein metabolism. Moreover, amla fruit extract with other polyherbal ingredients can improve immunity and udder health in cows [32]. Furthermore, Rizwan et al. [33] have reported that *Phyllanthus emblica* fruit extract is an inexpensive way to treat subclinical mastitis, and it can also be used to substitute antibiotic therapy to produce antibiotic residue-free milk. Almatroodi et al. [34] reviewed that amla fruit can be used as a natural feed additive in health management and this has been proven through its antioxidant, anti-inflammatory, hepatoprotective, gastroprotective, anti-diabetic, anti-microbial, neuro-protective, cardioprotective, and immunomodulatory activity [34].

To our knowledge, no studies have previously investigated the effects of fresh amla fruit on blood metabolites, antioxidant capacity, and milk FA profiles in lactating cows. Thus, the objective of this study was to investigate the effect of a diet supplemented with fresh amla fruit as a natural feed additive on blood metabolic parameters, milk FA profiles, and milk and blood oxidative capacities in lactating dairy cows.

## 2. Materials and Methods

### 2.1. Fresh Amla Fruit Extraction, Characterization, and Quantification of Metabolites

In this study, we adjusted the extraction, characterization, and quantification of metabolites based on Wang et al. [35], as briefly stated below.

#### 2.1.1. Amla Fruit Sample Preparation and Extraction

Samples of fresh amla fruit were freeze-dried and crushed with a zirconia bead in a mixer mill for 1.5 min at 30 Hz. The sample was extracted by mixing 100 mg of lyophilized powder with 1.2 mL 70% methanol solution, vortexed for 30 s every 30 min for six times and kept in a refrigerator overnight. The extracts were centrifuged at 12,000 rpm for 10 min before filtration followed by UPLC-MS/MS analysis.

UPLC-ESI-MS/MS was used to analyze sample extracts (UPLC, SHIMADZU Nexera X2, available at http://www.shimadzu.com.cn/, accessed on 1 January 2022; MS, Applied Biosystems 4500 Q TRAP, available at http://www.thermofisher.cn/cn/zh/home/brands/applied-biosystems.html, accessed on 1 January 2022).

#### 2.1.2. ESI-Q TRAP-MS/MS

To acquire the linear ion trap (LIT) and triple quadrupole scans, an AB4500 Q TRAP UPLC/MS/MS System was used, equipped with an ESI Turbo Ion-Spray interface, running both in positive and negative ion mode and controlled by Analyst 1.6.3 software (AB Sciex, Warrington, UK). The triple quadrupole (QQQ) scans were acquired using collision gas (nitrogen) at a medium setting in multiple reaction monitoring (MRM) experiments. Further optimization of declustering potential (DP) and collision energies (CE) was performed for individual MRM transitions.

#### 2.1.3. Characterization and Quantification of Metabolites

According to the self-built database MWDB (metware database), the material was characterized according to secondary spectral information. During the analysis, the repeated signal containing K^+^ ions, Na^+^ ions, NH_4_^+^ ions, and fragmented ions bearing other large molecular weights were removed. The number of metabolites in samples were determined using the MRM mode of QQQ mass spectrometry. Finally, based on the relative contents of the samples, we selected the top 15 metabolites from the phenolic acid, flavonoid, or tannin to detect metabolites based on peak time for the integral correction chart of metabolites (Table S3).

### 2.2. Animals, Diets, and Experimental Design

At the start of the experiment, eight ruminally cannulated Chinese Holstein mid-lactation dairy cows from 1 to 4 parity (mean ± SD: 2.13 ± 1.25 of parity; 634 ± 67.6 kg of average body weight (BW); 102.1 ± 4.49 days in milk (DIM) and 19.4 ± 2.59 kg/d of milk yield) were handled according to the guidelines of Institute of Animal Science (IAS, No. IAS20180115). Cows were housed in an individual stall barn and had free access to water. The eight cannulated lactating cows were randomly assigned to one of two treatments (control or supplemented) in a repeated crossover design. For all groups, we used the same total mixed ration (TMR) diet (Table 1 and Table 2, previously used from Tilahun et al. [28]), formulated using AMTS Cattle Pro version 4.14 (2018, AMTS LLC, Groton, NY, USA) and prepared using a feed mixer on a daily basis based on cows’ nutritional needs to produce 25 kg milk daily with a target of 3.5% milk fat and 3.1% milk protein. 

Experimental treatments were: (1) control—TMR with no amla fruit (0 g/day); (2) supplemented group—TMR supplemented with fresh amla fruit (FAF) administered as a top dress, in sequential order at three dose levels (200 g/d, 400 g/d, or 600 g/d, as-fed basis) (Table 3). Cows were fed twice daily at 07:30 and 15:30 h and allowed to refuse 5 to 10% of the TMR during the feeding sessions to receive ad libitum feed. Simultaneously, we added half of the daily allotment of FAF as a top dress to the diet at 07:30 and the other half at 15:30 h each day. This experiment was conducted between January and May 2020. Each period consisted of a cow as the main plot and three different FAF doses as subplots. After 14 d of the adaptation phase, period 1 of the feeding experiment started. In Period 1, four cows in the first group were fed the control diet plus each dose of added supplementary FAF feed (200, 400, and 600 g/d) sequentially, with each dose being supplemented for 14 d before increasing to the next dose. On the other hand, the remaining four cows were fed the control diet. Period 2 began after 14 d of the washout period, and the control and supplemented groups were exchanged (Table 3), as previously used in the infusion study by Wang et al. [36] in our laboratory, and Drackley et al. [37]. During each 14-day sub-period, the first 10 days were adjusted for diet adaptation, and the last four days were assigned to collect milk, feed, and blood samples.

### 2.3. Blood Sampling, Measurement, and Analysis

In each experimental period, at 0 h before morning feeding, blood samples from coccygeal vessels were collected, stored over ice, and transferred to the laboratory within one hour on days 11 and 13 of each subperiod. Vacutainer tubes without clot activator and with anticoagulant heparin fat were used to obtain serum and plasma, respectively, after centrifugation was performed at 2000× *g* for 20 min at 4 °C and stored at −20 °C until further investigation. The serum and plasma blood metabolites, which included enzymatic activities of alanine aminotransferase (ALT; U/L), aspartate aminotransferase (AST; U/L), uric acid (µmol/L), and lactic acid (LD, mmol/L) in addition to concentrations of glucose (mmol/L), total protein (g/L), albumin(g/L), globulins(g/L), albumin/globulin (A/G ratio), total cholesterol (mmol/L)), triglycerides (mmol/L), non-esterified fatty acid (NEFA, mmol/L), β-hydroxybutyrate (BHB, mmol/L), and creatinine (µmol/L), were measured using commercial kits from Nanjing Jiancheng Bioengineering Institute, Nanjing, China. Procedures were carried out according to the kit’s instructions. Globulin (GBL, g/L) content was estimated by the difference between total protein and albumin contents.

The activities of superoxide dismutase (SOD, U/mL), nitric oxide (NO, µmol/L), and the contents of Malondialdehyde (MDA, nmol/mL) in plasma were determined using colorimetric methods with a spectrophotometer using the Nanjing Built-in Kits (www.njjcbio.com, accessed on 11 February 2021) according to the manufacturer’s instructions, Nanjing Jiancheng Bioengineering Institute, (Nanjing, Jiangsu, China). Total antioxidant capacity (TAC, mM), ferric reducing-antioxidant power (FRAP, mM), and ascorbic acid (mM) content in milk was determined using colorimetric methods with a spectrophotometer using Biovision kits.

### 2.4. Milk Fatty Acid Composition

Milk samples were taken on days 12 and 14 at morning and afternoon milking’s of each subperiod to determine the milk fatty acid composition. Milk fatty acids profiles were carried out using the method described by Bligh and Dyer [38] and Bu et al. [39] with some modifications. Briefly, 200 μL milk samples of the frozen milk were thawed and homogenized with 1.5 mL water, 4 mL methanol, and 2 mL chloroform. The solution was re-homogenized with 2 mL chloroform, and following which, a weak salt solution of 2 mL of 1 N NaCl was added after being vortexed with a multi-vortexer for 30 min. Again, the tubes were vortexed for 2 min and centrifuged for 10 min at 3500 rpm. Following centrifugation, the entire lower phase was transferred gently with disposable plastic scale straw into a new 10 mL tube, evaporated to dryness in a nitrogen evaporator, and equipped at 45 °C with a thermostatically controlled water bath.

After adding 2 mL NaOH-CH_3_OH to screw cap glass tubes, the lipid sample was saponified and incubated in a 60 °C water bath for 5 min. We added 1 mL of boron trichloride in MeOH after cooling and then incubated it at 100 °C for 10 min, then 2 mL of heptane was added and incubated at 100 °C for 1 min. In the next step, 2 mL solution of saturated NaCl was added, and the mixture was centrifuged for ten minutes at 3500 rpm. Following centrifugation, the heptane layer was transferred into a 16 × 100 mm tube. Once again, the original tubes were filled with 2 mL of heptane, incubated, centrifuged, and the heptane layer extracted was transferred. By blowing with N_2_ on a heating block at 45 °C, transferred samples were evaporated. The final step was to reconstitute lipid extracts with 100 µL of heptane and transfer them to GC vials for chemical analysis.

A flame ionization detector was fitted to an Agilent 6890 GC (Agilent, Agilent Technologies, Santa Clara, CA, USA) to separate and analyze fatty acid methyl esters (FAMEs) quantitively. The samples containing methyl esters in hexane (2 μL) were injected through the split injection port (50:1) onto an HP-88 fused silica 100 m × 0.25 mm column, 0.20 µm film (Agilent, Agilent Technologies). The initial oven temperature, set at 120 °C for 10 min, was raised to 230 °C at a rate of 1.5 °C/min, and this temperature was maintained for 30 min. During the experiment, the injector temperature was held at 250 °C, the detector temperature was maintained at 280 °C, and the entire run duration was 113.33 min. The total amount of fatty acids was obtained by measuring the peak areas of the fatty acids on the GC readout, and then reported as g of fatty acid per 100 g of FAME.

To evaluate the nutritional quality of the lipid fraction of the milk samples, the atherogenicity index (AI) and hypocholesterolemic and hypercholesterolemic (h/H) ratio was calculated according to Rafiee-Yarandi et al. [11], while spreadability index (SI) was calculated based on Drackley et al. [37].

AI = [(C_12:0_ + 4 × C_14:0_ + C_16:0_)/(∑UFA)]

h/H = (C_18:1_ + PUFA)/(C_12:0_ + C_14:0_ + C_16:0_).

SI = C_16:0_/C_18:1_ cis-9

### 2.5. Determination of Total Flavonoids

The total flavonoid content was determined using a colorimetric aluminum chloride assay technique described by Bizuayehu [40]. An aliquot (200 µL) of milk sample was put to a 10 mL test tube containing 5 mL of distilled water. After 5 min, 250 µL of 5% NaNO_2_ was added to the tubes, followed by 500 µL of 10% AlCl_3_ and 2 mL of 1 M NaOH at the 6-min mark. The total volume was increased to 10 mL with distilled water. Distilled water was added to the total volume to make it 10 mL. After mixing well, the absorbance of the mixture at 510 nm was measured against the prepared reagent blank using a spectrophotometer. The total flavonoid concentration was calculated using a calibration curve and reported as mg quercetin equivalents per mL of milk (mg QT/mL). The calibration curve was prepared with 50, 100, 200, 400, 600, 800, and 1000 µg/mL. A standard curve of quercetin (mg/mL) was plotted with the equation y = 0.055x + 0.003, R^2^ = 0.9956, where y was the absorbance at 510 nm and x was the sample concentration in mg/mL. The determination was made in duplicate. The milk samples were diluted two-fold (1:1) based on preliminary experiments.

### 2.6. Statistical Analysis 

Data were analyzed by using PROC MIXED of SAS (version 9.4; SAS Institute Inc., Cary, NC, USA). The model contained the random effect of the cow and the fixed effects of the period (i.e., the six-week sets of experimental periods within each treatment type; 1 df), treatment (control or amla supplementation; 1 df), FAF dose supplemented (representing dose levels of 0, 200, 400, and 600 g/d as-fed basis, as a subplot; 3 df), and the interaction of treatment and amla dose (3 df) (Table 3). The control cows were fed only TMR for the whole week.

In order to avoid concerns regarding how cows would respond to sudden and large dose changes, amla fruit supplementation could not be randomized within Periods 1 and 2, and was therefore supplemented sequentially as in the study by Drackley et al. [37], and Wang et al. [36]. Due to this, the amla dose supplementation had a confounding effect on the time (weeks). Therefore, this study’s main statistical parameter of interest was the interaction between treatment and dose level [36,37] to determine whether cows treated with amla fruits responded differently with advancing time (i.e., weeks) compared with controls. In the analysis, we used the covariance structure that yielded the lowest Akaike’s information criterion to examine within-subject variation. The linear, quadratic, and cubic effects of dose level by treatment were divided into single degree of freedom interactions by using polynomial contrasts, and these contrasts’ *p*-values were calculated and summarized. In this case, the Kenward–Roger method was used to calculate degrees of freedom [41]. A pairwise correlation was evaluated to determine the interrelation between the milk fatty acid profiles or summation of fatty acid groups using JMP Pro 14. All calculations were presented with their standard errors and the least-squares means. The significance level was established at *p* ≤ 0.05, and trends were examined at 0.05 < *p* ≤ 0.10 levels. 

The principal component analysis (PCA) was employed to determine the effect of an amla dose on milk fatty acid composition. Before completing the PCA, the data were auto-scaled. The PCA was carried out with the FactomineR package (v2.4), the Factoshiny package (v2.4), and R Studio (v4.1.1) for analysis.

## 3. Results

### 3.1. Characteristics of Fresh Amla Fruit

**Figure 1:**
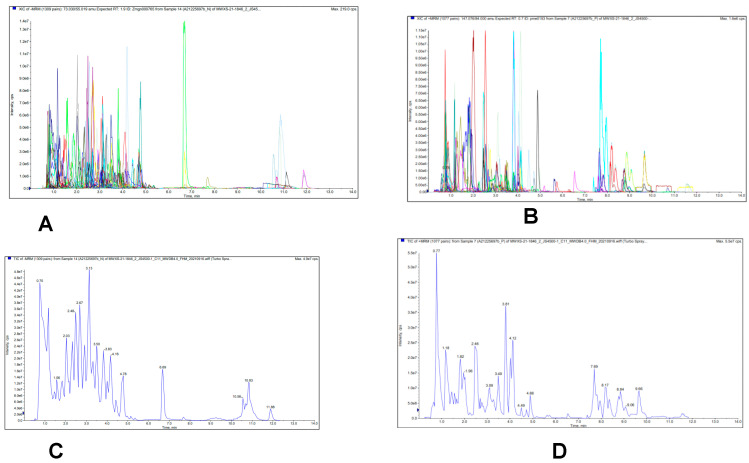
(**A**,**C**) Multiple reaction monitoring (MRM) and total ions current (TIC) and metabolite detection of negative ion mode; (**B**,**D**) MRM and TIC metabolite detection of positive ion mode multipeak mass spectral chromatogram of metabolites of fresh amla fruit.

The metabolites of fresh amla fruit were identified using UPLC-ESI-MS/MS. Total ions current (TIC) and MRM metabolite detection multi-peak graphs of the fresh amla fruit are shown in Figure 1. A total of 514 metabolites were detected, including: 113 phenolic acids, 102 lipids, 101 flavonoids, 46 amino acid and derivatives, 38 tannins, 19 alkaloids, 16 organic acids, 16 terpenoids, 12 lignans and coumarins and derivatives, 78 amino acids, and derivatives, 66 phenylpropanoids, 61 lipids, 57 nucleotides and derivates, 40 alkaloids, 27 terpenes, 22 carbohydrates, 18 vitamins and derivatives, 10 nucleotides and derivatives, and 41 others (Tables S1 and S2).

**Figure 2:**
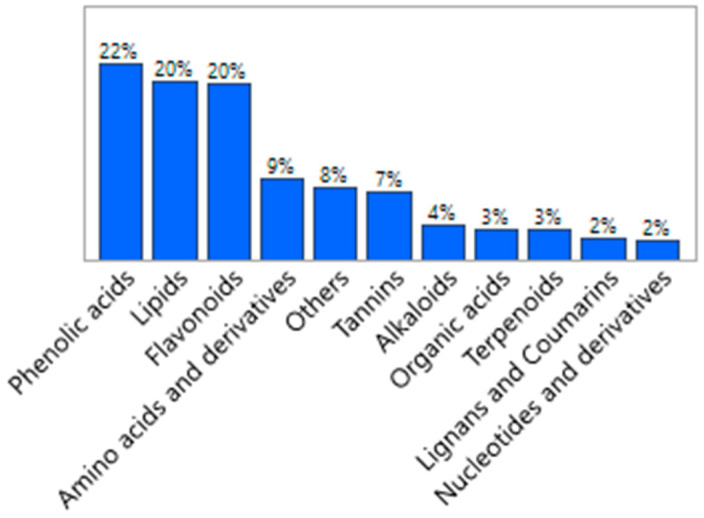
Classification of detected metabolites in fresh amla (*Phyllanthus emblica*) fruit.

There were five main metabolic products: phenolic acids (22%), flavonoids (20%), lipids (20%), amino acids and derivatives (9%), and tannins (7%) (Figure 2). This study provides essential basic information on metabolites of amla fruit for further research.

### 3.2. Milk Fatty Acid Profiles

The fresh amla fruit supplementation had a noticeable effect on the fatty acid (FA) profile of milk, and its effects on the composition of milk fat (g/100 g of total FA) are shown in Table 4. The contents of the C_10:0_ and C_15:0_ fatty acids increased in a cubic fashion (*p* < 0.001, 0.03, respectively) and a quadratic manner (*p* < 0.01, 0.05, respectively) with increasing doses of fresh amla fruit. Compared with controls, fat milk profiles increased a little at 200 g/day, decreased sharply when the dosage increased to 400 g/day, and then increased dramatically when the dose increased to 600 g/day FAF. The milk FA profiles of C_12:0_ (lauric acid), C_14:0_ (myristic acid), and C_16:0_ (palmitic acid) responded cubically (*p* < 0.001, 0.01, 0.03, respectively) with increased FAF dose, and were reduced at 200 or 400 g/d but increased at 600 g/d FAF dose compared to controls. The total sum of saturated fatty acids (SFA) and health index parameters, such as the atherogenicity index (AI), were reduced at 200 or 400 g/d FAF doses in a quadratic way (*p* < 0.001, <0.01, respectively) compared to controls. Conversely, amla fruit supplementation improved the milk FA contents of *anteiso* C_17:0_ (*p* < 0.001), C_18:1_ cis-9 (oleic acid, OA, *p* < 0.01), the biohydrogenation intermediates (C_18:1_ trans-11, vaccenic acid, VA, tended, *p* = 0.08), C_24:1_ (*p* < 0.01), C_20:5_ (Eicosapentaenoic acid, EPA, *p* = 0.03), and C_22:6_ (Docosahexaenoic acid, DHA, *p* < 0.01,) quadratically at 200 or 400 g/d FAF dose compared to controls. The increased proportion in most omega-3 PUFA such as EPA and DHA resulted in a decreased omega-6/omega-3 ratio and increased the health index parameter ratio of hypocholesterolemic and hypercholesterolemic (h/H) quadratically (*p* < 0.001), compared to controls. In addition, the milk fat contents of C_17:0_, C_18:0_ (stearic acid, SA), C_20:0_, C_22:0_, and C_22:2_ were increased when amla fruit doses were supplemented, with increases being greater when the 400 g/d FAF dose was supplemented compared to controls (quadratic effect, *p* < 0.01, <0.001, 0.04, 0.02, <0.001, respectively). Furthermore, the total proportion of branched chain FA (BCFA), monounsaturated FA (MUFA), polyunsaturated FA (PUFA), and unsaturated FA were increased quadratically (*p* < 0.001, 0.02, <0.001, respectively) at 200 or 400 g/d and reduced at 600 g/d FAF dose compared to controls. The above results showed that the inclusion of FAF at 400 g/d was more effective in increasing the concentration of beneficial FA compared with control groups. Moreover, FAF supplementation reduced the butter spreadability index (SI) quadratically (*p* < 0.01), with the lowest value recorded at 400 g/d FAF dose, and it was reduced by 39.2% compared to controls.

The correlation results are reported in Table S4. The correlation results showed total saturated FA (SFA) was positively correlated with poor health indicators C_12:0_ (r = 0.63, *p* < 0.001), C_14:0_ (r = 0.82, *p* < 0.001), C_16:0_ (r = 0.86, *p* < 0.001), and AI (r = 0.92, *p* < 0.001), while it was negatively correlated with beneficiary FA such as C_18:0_ (r = −0.54, *p* < 0.001), EPA(r = −0.47, *p* = 0.001), DHA (r = −0.56, *p* < 0.001), MUFA (r = −0.79, *p* < 0.001), UFA (r = −0.86, *p* < 0.001), and h/H (r = −0.87, *p* < 0.001). In addition, the health index indicators AI and h/H were negatively correlated (r = −0.92, *p* < 0.001). Moreover, the butter quality indicator SI was positively correlated with C_12:0_ (r = 0.47, *p* = 0.001), C_14:0_ (r = 0.60, *p* < 0.001), C_16:0_ (r = 0.89, *p*< 0.001), SFA (r = 0.89, *p* < 0.001), and AI (r = 0.89, *p* < 0.001). However, SI was negatively correlated with SA (r = −0.83, *p* < 0.001), MUFA (r = −0.89, *p* = 0.001), UFA (r = −0.90, *p* < 0.001), and h/H (r = −0.86, *p* < 0.001).

### 3.3. Exploring Milk Fatty Acid Data with PCA

**Figure 3:**
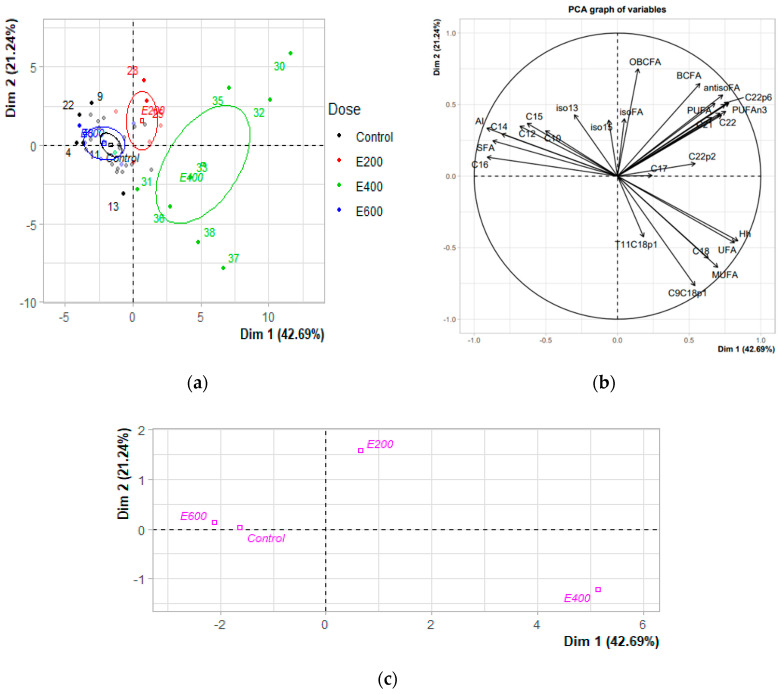
(**a**) Individuals factor map (PCA); (**b**) variable’s factor map (PCA); (**c**) qualitative factor map (PCA). The labeled factors are those the best shown on the plane, in which: control (0 g/d FAF); E200 (200 g/d FAF as fed); E400 (400 g/d FAF as fed); and E600 (600 g/d FAF as fed).

The dataset was subjected to PCA to investigate more thoroughly possible relationships between fatty acid composition and amla fruit diet (Figure 3a–c). The results of the PCA for the fatty acids revealed the first four principal components (PC), which explained 80.97% of the total variability in fatty acid composition with the amla fruit supplementation study (Table 5). PC1 (Dim1) explained 42.69% of the total variability in fatty acid composition, PC2 (Dim2) explained 21.24% of that variability, and PC3 and PC4 together accounted for 17.03% of the total variability. The PCA separated dairy cows into four distinct clusters according to feed group (Figure 3c). PCA results were shown in figures to compare the score plot (doses: Figure 3a) with the loading plot (milk fatty acids: Figure 3b). PC1 describes 42.69% of the variance in the dataset, and its loadings indicate that it has high contributions from C_16:0_ (palmitic acid, 0.91), atherogenicity index (AI, 0.91), total saturated FA (SFA, 0.87), hypocholesterolemic and hypercholesterolemic ratio (h/H, 0.84), unsaturated FA (UFA, 0.82), and C_14:0_ (Myristic acid, 0.81), omega-3 PUFA (0.78), C_22:6_ (DHA, 0.78), C_20:0_ (0.76) *anteiso* C_17:0_ (0.75) variables. Whereas palmitic acid, AI, SFA and myristic acid exhibited negative loadings, h/H, omega-3PUFA, DHA, C_20:0,_ and *anteiso* C_17:0_ had positive loadings denoting the significant relationship of their contributions to the data variability. PC2 showed a high negative loading for C_18:1_ cis-9 (OA, 0.77) and positive loadings for odd and branched-chain FA (OBCFA, 0.75) and branched-chain FA (BCFA, 0.65) variables.

The objective of a loading projection is to visualize the position of the variables with respect to one another in a two-dimensional space and their corresponding correlations. Variables closest to one another and far from the plot origin are positively correlated (or directly proportional, e.g., see SFA and C_16:0_ in the Dim1–Dim2 plot), while variables opposite one another on the plot are negatively correlated (or inversely proportional, e.g., see UFA and h/H in the Dim1–Dim2 plot). From the figure, the difference between the samples from cows with no amla fruit supplementation in their diet and the samples from cows with fresh amla fruit in the diet is apparent. From the comparison of the PCA score plot in Figure 3a and the PCA loading plot in Figure 3b, it can be interpreted that the content of C_18:1_ cis-9 (OA), C_18:0_ (SA), UFA, MUFA, h/H, C_22:0_, BCFA, *anteiso* FA, PUFA, EPA was higher in the groups of 400 g/d amla dose supplementation, whereas the content of C_16:0_, SFA, AI, C_14:0_, and C_12:0_ was higher in the milk samples of groups fed control or 600 g/d amla fruit supplementation. The above PCA result also agrees with the statistical analysis reported in Table 4.

### 3.4. Blood Biochemical Parameters

The effect of fresh amla fruit supplementations on blood metabolites and liver enzymes are shown in Table 6. Beta-hydroxybutyrate (BHB), non-esterified fatty acid (NEFA), and triglyceride in serum and creatine, and uric acid concentrations in plasma were not affected by feeding amla fruit doses (*p* > 0.23). In addition, amla fruit supplementation did not affect liver enzymes in serum, including alanine amino-transferase, aspartate aminotransferase, and Gamma-glutamyl transferase (*p* > 0.10).

However, supplementing amla fruit for lactating cows increased serum glucose at all amla doses quadratically (*p* < 0.01), with increases greater when 400 g/d FAF dose was supplemented compared with controls. In addition, total serum cholesterol (*p* = 0.01), total protein (*p* < 0.001), and globulin (*p* < 0.001) responded quadratically, and increased concentration at 200 or 400 g/d amla fruit dose, but reduced at 600 g/d compared to controls. Consequently, the albumin: globulin ratio was responded linearly (*p* < 0.001) and in a cubic way (tended, *p* = 0.06) and reduced at 200 g/d, but increased sharply starting from 400 g/d amla fruit dose compared to controls. The plasma lactic acid concentration increased cubically (*p* = 0.04) with increasing amla fruit doses up to 400 g/d, and after that, the concentration was reduced at 600 g/d amla fruit dose compared to controls. 

### 3.5. Antioxidant Capacity in Milk and Plasma

The effect of fresh amla fruit supplementations on blood plasma and milk antioxidant capacities are shown in Table 7. The blood plasma antioxidant enzyme malondialdehyde (MDA, *p* = 0.57) was not affected with amla fruit supplementation. However, plasma nitric oxide (NO) responded linearly (*p* < 0.001), and in a quadratic way (*p* = 0.04), increasing first at 200 g/d (+13.1%), reducing slightly at 400 g/d (−11.0%), and then sharply reducing at 600 g/d (−39.9%) of amla supplementation compared to controls. Conversely, superoxide dismutase (SOD) levels in plasma responded linearly (*p* = 0.01) and cubically (*p* < 0.01) and were reduced at 200 g/d (−7.7%) but increased at 400 g/d (+14.0%) or 600 g/d (+6.2%) amla fruit doses compared to controls. Similarly, ferric reducing-antioxidant power (FRAP) antioxidant capacity in milk was reduced first at 200 g/d and then increased at 400 g/d, but reduced again at 600 g/d in a cubic fashion (*p* < 0.001) compared to controls. However, the total antioxidant capacity (TAC, linear, *p* < 0.01) was increased at all dose levels compared to controls. Moreover, flavonoid, and ascorbic acid, which influence amla fruit’s antioxidant capacity, were transferred to milk and increased quadratically in milk (*p* < 0.001, 0.01, respectively) with alma fruit supplementation with the most remarkable increment recorded at 400 g/d amla fruit doses compared to controls.

## 4. Discussion

### 4.1. Fresh Amla Fruit Characteristics

Similar to our study, Mao et al. [42] regarding the Phyllanthus plant reported 510 compounds, including lignans, triterpenoids, flavonoids, and tannins. However, Wu et al. [43] detected lower metabolites from amla using UPLC-MSn, indicating 110 compounds with dominant hydrolyzable tannins 41% (45/110), phenolic acids 14% (15/110), flavonoids 14% (15/110). Khaled et al. [44] performed UPLC-qTOF-MS analysis for P. emblica and determined that hydrolyzable tannins dominated the analysis (37/92), while flavonoids accounted for (2/92). Moreover, Yang et al. [25] detected 144 peaks of which 67 had tentatively been identified as ellagitannins, flavonoids, and simple gallic acid derivatives in the fractions. In contrast, in this study the dominants were phenolic acid (22%) and flavonoids (20%). The variation may be related to the method of analysis, environmental, and genetic factors.

### 4.2. Milk Fatty Acid Profile

Milk fatty acid (FA) profile determines the physical and organoleptic properties of milk, which affect the quality of milk and milk products and the nutritional properties of milk through specific FA impacts on consumer health [45]. Milk fat proportions of C_12:0_, C_14:0_, and C_16:0_, synthesized *de novo* in the mammary gland, were decreased dose-dependently at 200 or 400 g/d, but increased at 600 g/d FAF dose. Interestingly, fatty acid C_18:0_ was increased at 200 and 400 g/d amla fruit levels. Excessive consumption of milk fat concentrations of C_12:0_, C_14:0_, and C_16:0_ fatty acids in human diets is a recognized risk factor for cardiovascular disease and can lower insulin sensitivity, while C_18:0_ is considered as having neutral effects on circulating plasma cholesterol concentrations [46]. Conversely, total proportion of MUFA, PUFA, and UFA were increased at 200 or 400 g/d FAF dose with the greatest increment measured at 400 g/d (+24.3, +35.4, and +26.4%, respectively) which reduced at 600 g/d (−5.1, −9.9, and −5.8%, respectively) compared to controls. These results are similar to those reported by Buccioni et al. [47] and Valizadeh Yonjalli et al. [48]. Therefore, the high concentration of UFA in the milk at 200 or 400 g of amla fruit supplementation might be related to the effect of amla polyphenols on the rumen microbial population involved in ruminal biohydrogenation.

Supplementation with amla fruit decreased health-related lipid indices such as the atherogenic index (AI) and omega-6 PUFA/omega-3 PUFA ratio, and increased hypocholesterolemic and hypercholesterolemic (h/H). The h/H index is the opposite of AI, because the higher the h/H is, the better nutrition is, and the lower the risk of cardiovascular disease. According to Addis, Cabiddu, Pinna, Decandia, Pirisi and Molle [12], fat with a high AI value is harmful to human health. The reduction in AI value by 12.5 and 40% and the increment of h/H ratio by 11.9 and 75% at 200 or 400 g/d amla dose, respectively, indicate a beneficial impact of amla fruit on reducing the risk of cardiovascular disease and metabolic diseases. In addition, at 200 or 400 g/d doses, amla fruit increased milk branched-chain fatty acids (BCFAs), which possess several benefits for human health, such as improving pancreatic β-cell function and inhibiting tumor growth [49,50]. Since BCFAs are derived largely from the membranes of rumen bacteria, the increase in this FA in milk from cows fed amla fruit was likely due to the changes in the bacterial populations [51].

Moreover, nutritionally rich omega-3 FA such as C_20:5_ (Eicosapentaenoic acid, EPA) and C_22:6_ (Docosahexaenoic acid, DHA) was increased at 200 or 400 g/d FAF dose and improved most at 400 g/d by 333.3 and 114.3%, respectively compared to controls. Consequently, changes in these parameters demonstrated that supplementation of amla fruit up to 400 g/d improves the nutritional quality of milk for humans by reducing the risk of health problems. The negative effect of 600 g/d of amla fruit was unexpected, and we suspect it might be related to some modifications in microbes, which requires further study. Similar to our study, the addition of coffee peel fermented flour in the feed significantly increased EPA and DHA level depositions in the fish fillet [52]. In addition, Abarghuei et al. [53] and Szczechowiak et al. [54] reported pomegranate-peel extract and condensed tannins and oil blends increased both EPA and DHA in the milk of dairy cows.

Ruminal biohydrogenation (BH) is characterized by biochemical reactions wherein PUFAs, mainly C_18:2_ cis-9, cis-12, and C_18:3_ cis-9, cis-12, cis-15, are isomerized and subsequently saturated progressively, predominantly by gram-positive ruminal bacteria, to produce stearic acid (C_18:0_) as the final product [55]. Most reports using condensed tannin as a feed additive for dairy cows reported decreases in C_18:0_ related to the inhibition of rumen BH [53,56]. However, in agreement with our study, Buccioni et al. [57] and Aprianita et al. [58] reported an increase in the content of milk fat C_18:0_, which indicates completion of the ruminal BH process to a certain extent, and the inhibition effect on C_12:0_ and C_14:0_ when supplemented with tannin extract from a chestnut (hydrolysable tannin source). Most research demonstrated that fat content of C_18:0_ was increased when animals were fed hydrolysable tannin source feeds. Further studies need to examine hydrolysable tannin impacts on the concentrations of milk fat C_18:0_ as well as the holistic biohydrogenation mechanism and mammary metabolism related to the tannin source.

Ruminal biohydrogenation (BH) is affected by condensed tannins and could be used as a potential means to increase rumenic (RA; C_18:2_ cis-9, trans-11) and vaccenic acid (VA; C_18:1_ trans-11) content in foods of animal origin [5]. In the current study, the proportion of VA, a precursor to CLA, MUFA, PUFA, and UFA, was increased in the milk FA profile at 200 or 400 g/d FAF supplementation. In addition, the increase in oleic acid (OA, C_18:1_ cis-9) at 200 or 400 g/d amla dose could have resulted from the partial biohydrogenation of C_18:2_ and C_18:3_ FA rumen [59]. Moreover, increasing the oleic acid content improves the spreadability of butter at refrigerator temperature [37]. Several researchers have reported that distinct bioactive compounds affect biohydrogenation (BH) at the specific inhibition of the last BH step (i.e., the saturation of trans 18:1 to 18:0), which increases VA concentration [60]. The advantage of increasing the availability of VA through the action of stearoyl CoA desaturase in the mammary gland would promote the synthesis of CLA [18]. Another scenario demonstrated a rate reduction in the initial biohydrogenation steps, favoring the ruminal accumulation of the PUFA diet [61]. This study’s enhancement of milk fat VA, OA, and PUFA showed that biohydrogenation inhibition was achieved at the initial and final BH stages when amla fruit was supplemented at the 200 or 400 g/d doses. In contrast, some studies have shown that tannin source feeds have not affected VA concentration [62]. These variations may be due to bioactive, structural, and chemical dissimilarities, making it difficult to generalize these phenolic compounds [61]. Furthermore, as reported by Buccioni et al. [57] and Alipanahi et al. [63], the increased total UFA concentration in the milk of cows fed amla fruit-containing diets may be attributable to the inhibitory effect of tannins on the growth of cellulolytic bacteria, including *Butyrivibrio fibrosolvens* and *Butyrivibrio proteoclasticus*.

### 4.3. Blood Biochemical Parameters

Polyphenols, tannins, flavonoids, and ascorbic acid are the primary antioxidants and bioactive components found in amla fruit [19,21,22]. Serum albumin is a multi-functional protein that can bind and transport numerous endogenous and exogenous compounds [64], and is also believed to act as an antioxidant because of free radical scavenging to protect bound substances from peroxidative damage, such as FA and lipoproteins [65]. In this study, amla fruit affects total protein, albumin, and globulin in a dose-dependent manner. The results showed that the optimum amla fruit dose of supplementation should not exceed 400 g/d, since 600 g/d amla fruit supplementation caused a decrease in the concentration of total protein, albumin, and globulin, which may be related to the availability of the dominant hydrolyzable tannins (HTs) in amla fruit [25], which may have toxic effects and lead to systemic inflammation or microbial adaptation. Some studies have indicated that HTs and/or their intermediary metabolites may be harmful to the liver [66]. However, different studies on amla fruit toxicity have shown that the fruit did not cause acute or chronic toxicity in rats [67,68], which may require further study if there are toxicity effects in ruminant animals. The highest increase in albumin, total protein, and globulin at 400 g/d could indicate amla fruit’s positive effect on improving protein utilization efficiency, and may improve liver function. Moreover, our previous result using the same experiment animals also showed increased milk nitrogen efficiency and milk protein, while reducing NH_3_-N, milk urea nitrogen, and protozoa population counts at 400 g/d FAF dose [28]. Similarly, buffalo calves supplemented with phytogenic feed additives rich in tannins, saponins, and essential oils containing fruit pulp of amla as one component found higher values for total protein, albumin, and globulin compared to the control group [29]. Moreover, biochemical parameters such as total protein, globulin, and glucose were increased when supplemented with bee propolis extract [69].

In ruminants, glucose net availability mainly depends on gluconeogenesis, and the main glucogenic nutrients are glucogenic amino acids, propionate, and lactate [70]. In addition, the glucose metabolism in the lactating dairy cow represents a balance between glucose requirement for milk production and glucose precursors supply from the diet, which is integrated by the liver as the most important endogenous gluconeogenesis organ [71]. In this study, amla fruit supplementation caused an increase in serum glucose at all dose levels of 15.4, 18.3, and 6.3%, for 200, 400, and 600 g/d amla fruit dose supplementations, respectively, compared to controls, demonstrating the potential to enhance gluconeogenesis. Similarly, when goats were fed dietary tea catechins, their plasma glucose levels were higher for all treatment groups than for the controls [72]. In addition, Szczechowiak et al. [54] reported that using a mixture of condensed tannins (*Vaccinium vitis idaea*) and oils blends for dairy cows significantly increased blood glucose levels. Increased blood glucose in this experiment may have been the consequence of elevated propionate production or organic matter (OM) digestibility for 200 or 600 g/d FAF dose from our previous published result [28]. Moreover, the high glucose concentration for 400 g/d FAF dose in the current experiment was probably due to greater amounts of absorbed amino acids in the gastrointestinal tract of animals, as noted by Zhang et al. [73], which needs to be further investigated. 

Amla fruit supplementation caused an increasing trend in total cholesterol concentrations in blood plasma at 200 and 400 g/d FAF doses, but did not exceed dairy cow reference values [74]. Similarly, Singla et al. [75] reported an increased trend in blood cholesterol concentration for buffaloes supplemented with *Emblica officinalis* fruit pomace than the control. In addition, the inclusion of CT from *Acacia mearnsii* extract in the lamb diets showed a trend of linear increment for the serum concentrations of cholesterol [76]. The reason for higher blood cholesterol concentrations from 200 to 400 g/d FAF supplementation and a reduction at 600 g/d in the amla fruit-fed dairy cows than the control cows is not clear based upon our results. 

### 4.4. Liver and Antioxidant Enzymes

Serum levels of AST, ALT, and ALP are valuable indicators of domestic animal hepatic damage [29]. In our study, feeding amla fruit up to 600 g/d doses did not affect lactating dairy cows’ AST, ALT, or GGT. However, AST showed a numerical reduction, and ALT and GGT obtained a numerical increment when supplemented with amla fruit doses. In addition, the value of GGT did not exceed the reference value for Holstein dairy cows [74]. Nitric oxide is a molecule regulating many biological functions in the body and plays an important role during the inflammatory process [77]. In this study, at the first dose of 200 g/d FAF supplementation, the NO concentration was increased, and SOD reduction at the first supplementation may have some toxic effects and form peroxynitrite, since research reports have shown that NO increases and reacts with superoxide anions during inflammation, leading to the formation of peroxynitrite radical [65]. However, amla fruit supplementation at 400 or 600 g/d FAF doses improved both NO and SOD, confirming that fresh amla fruit is an excellent antioxidant due to its inhibitory effects on reactive oxygen species’ (ROS) metabolism. Thereby, it can play an important antioxidant role in protecting cells from oxidative stress. However, further study is required to evaluate the effect of amla fruit on different oxidative stress environments. Similarly, an in vitro study by Chan et al. [78] reported the presence of 1% ethanol, quercetin, from 3 to 100 mM, that reduced NO production in a concentration-dependent manner by 21.8 to 99%, and speculated that ethanol and quercetin, both scavengers of free radicals, may reduce iNOS mRNA expression by preventing oxidative stress-induced action on the transcription factor NFkB. Moreover, Srivastava et al. [79] reported green tea polyphenols and tannic acid were most effective factors in inhibiting NO generation (90%).

### 4.5. Antioxidant Capacity and Status in the Milk

Amla fruit supplementation affected antioxidant capacity and status in milk in a dose-dependent manner. The highest potential in milk FRAP antioxidant capacity, flavonoid, and ascorbic acid (Vitamin C) were measured at 400 g/d FAF dose compared to controls. Serum albumin is also believed to act as an antioxidant and follow the same path as FRAP, with amla supplementation highest at 400 g/d FAF dose, compared to controls. Similar to this study, it was found that adding 9% and 18% of pelleted citrus pulp to regular diets increased total polyphenol and flavonoid concentration, and ferric reduced antioxidant power in cows’ milk [80]. Furthermore, when assessed by FRAP, milk extracts from goats fed under a conventional diet supplemented with 30% graded Acacia pod levels showed the best antioxidant capacity performance [81]. In contrast, feeding with pomegranate seed oil or linseed oil did not affect the concentrations of flavonoids in milk; however, feeding pomegranate seed oil sharply increased the total polyphenols of milk [82]. The different effects on phenols and flavonoids may be related to differences in plant source and species. The observed increase in FRAP values can be attributed to amla fruit bioactive components transferring into the bloodstream and milk via absorption from the digestive system [83]. Thus, these results suggest that it is possible to improve the antioxidant status of lactating cows by feeding them fresh amla fruit (optimum level, 400 g/d) as a natural antioxidant.

## 5. Conclusions

An analysis of fresh amla fruit with a mass spectrometry UPLC-ESI-MS/MS system revealed that it is rich in primary and secondary components such as phenolic acid, flavonoids, lipids, tannins, and others. Supplementation of amla fruit affected the milk fatty acid profiles, blood metabolites, and milk antioxidant capacity in a dose-dependent manner. Fresh amla fruit modulated biohydrogenation and produced more health-beneficial milk fatty acids at 200 or 400 g/d FAF dose supplementation. However, the most remarkable improvements in protein efficiency and antioxidant capacity in the blood (for serum albumin and plasma SOD, respectively) were recorded at the 400 g/d FAF dose compared to controls. Moreover, milk FA profiles such as total BCFA, UFA, PUFA, n-3 PUFA and MUFA, and individual FA proportions of DHA, EPA, OA, and VA proportions showed maximum improvements at 400 g/d amla fruit dose compared to controls. In addition, the health index parameters AI, h/H, and the n-6/n-3 PUFA ratio also verified the beneficial effect of amla fruit dose at 400 g/d for human consumption compared to controls. Furthermore, blood metabolites also indicated that amla fruit supplementation increased protein efficiency with increased serum albumin and total protein at 400 g/d FAF doses compared to the controls.

Furthermore, the plasma antioxidant biomarkers SOD and albumin, and the milk antioxidant capacities FRAP and TAC, verified the antioxidant capacity of amla fruit for lactating cows at 400 g/d FAF dose compared with controls. In addition, butter spreadability was also improved in cattle fed FAF at 400 g/d dose compared to controls. Therefore, supplementing fresh amla fruit doses for lactating cows at 400 g/d on an as-fed basis can be successfully used as an alternative additive feed in dairy cow diets to improve antioxidant capacity, protein efficiency, butter quality, and to produce healthier milk fatty acid profiles for human consumption. However, further investigation is required to verify the negative impacts of feeding 600 g/d FAF doses to lactating dairy cows.

## Figures and Tables

**Table 1:** Chemical composition of feed ingredients of the total mixed ration (TMR) used to feed lactating dairy cows (Least squares mean ± SE ^#^).

Item	Value
TMR	
Ingredient, g/kg DM	
Corn silage	347.6 ± 0.64
Alfalfa hay	152.5 ± 0.62
Concentrate mix ^1^	499.9 ± 0.06
Chemical composition (g/kg DM)	
Dry matter	488 ± 4.1
Ash	86.7 ± 6.41
Crude protein (CP)	164 ± 1.5
Neutral detergent fiber (aNDF)	291 ± 4.7
Acid detergent fiber	173 ± 5.4
Ether extract (EE)	14.4 ± 0.96
Non-fiber carbohydrates (NFC) *	451 ± 8.8
Acid insoluble ash	43.1 ± 4.92

^#^ Standard error; ^1^ 195.7 g/kg soya bean meal, 94.4 g/kg cottonseed meal, 41.0 g/kg canola meal, 102.4 g/kg DDGS, 21.6 g/kg corn gluten wet, 450.5 g/kg corn grain grind fine, 17.1 g/kg calcium carbonate, 10.2 g/kg salt (NaCl),6.8 g/kg manganese oxide, 8.0 g/kg calcium phosDi, 21.6 g/kg fat corn oil, 18.2 g/kg sodium bicarbonate, 12.5 g/kg vitamin premix ADE. * NFC, calculated according to NRC (2001); NFC, calculated as 1000 − (NDF + CP + EE + ash).

**Table 2:** Chemical composition of individual total mixed ration (TMR) ingredients and additive fresh amla fruit.

Item	Alfalfa Hay	Corn Silage	Concentrate Mixture	Amla Fruit	SE *
Chemical composition (g/kg DM unless noted)					
Dry matter	872	286	860	231	4.1
Ash	103	64	83	28	4.6
Crude protein (CP)	169	73.0	224	35.4	2.41
Neutral detergent fiber (aNDF)	387	413	172	347	5.2
Acid detergent fiber	275	257	83.6	237	7.63
Ether extract (EE)	8.13	7.07	30.4	6.44	1.422
Non-fiber carbohydrates ^1^	332	453	514	588	8.8
Total phenolic content (mg TA/g DM) ^2^	-	-	-	51.2	1.84
Total flavonoid content (mg QT/g DM) ^3^	-	-	-	87.8	1.63

* Standard error; ^1^ non-fibre carbohydrates, calculated as 100 − (aNDF + CP + EE + ash); ^2^ mg TA/g; milligram tannic acid/gram; ^3^ mg QT/g; milligram quercetin/gram.

**Table 3:** Schematic of experimental design and supplementation of fresh amla fruit doses.

Cow	Weeks							
	Adaptation	Period 1			Washout	Period 2		
	−2	2	4	6	8	10	12	14
	Fresh amla fruit (g/d)							
MY010	0	200	400	600	0	0	0	0
MY001	0	200	400	600	0	0	0	0
P71	0	200	400	600	0	0	0	0
090922	0	200	400	600	0	0	0	0
1108	0	0	0	0	0	200	400	600
MY002	0	0	0	0	0	200	400	600
P34	0	0	0	0	0	200	400	600
P72	0	0	0	0	0	200	400	600

**Table 4:** Milk FA ^1^ profile (g/100 g of total fatty acids) of lactating cows fed a TMR without supplementation (control, 0 g/d), or supplemented with 200, 400 or 600 g/d amla fruit doses.

Items	Fresh Amla Fruit Doses Supplemented g/day					Treatment by Dose, *p*		
	0	200	400	600	SE *	Linear	Quadratic	Cubic
Saturated FA (SFA)								
C_10:0_	0.67	0.72 (0.67) ^1^	0.15 (0.69)	0.98 (0.67)	0.13 0.15	0.45	<0.01	<0.001
C_12:0_	2.38	2.50 (2.70)	0.90 (1.90)	2.84 (2.43)	0.22 0.20	0.79	<0.001	<0.001
C_14:0_	9.67	8.99 (10.0)	6.38 (8.98)	10.0 (9.88)	0.50 0.40	0.47	<0.001	<0.01
C_15:0_	1.34	1.35 (1.33)	1.00 (1.41)	1.42 (1.27)	0.01 0.15	0.83	0.05	0.03
C16:0 (palmitic acid)	43.2	39.6 (41.7)	34.4 (44.9)	43.0 (42.5)	1.5 1.6	0.37	<0.001	0.03
C_17:0_	0.63	0.72 (0.66)	0.75 (0.64)	0.61 (0.57)	0.04 0.04	0.86	<0.01	0.53
C18:0 (stearic acid)	6.34	7.17 (6.34)	11.2 (6.00)	6.95 (6.63)	0.73 0.87	0.06	<0.001	<0.01
C_20:0_	0.20	0.43 (0.18)	0.89 (0.17)	0.39 (0.22)	0.17 0.02	0.19	0.04	0.16
C_21:0_	1.02	1.32 (0.87)	1.94 (1.01)	1.40 (1.16)	0.25 0.10	0.12	0.10	0.23
C_22:0_	0.29	0.57 (0.25)	0.93 (0.31)	0.44 (0.29)	0.17 0.07	0.27	0.02	0.25
C_23:0_	0.91	1.47 (0.94)	1.25 (0.88)	0.99 (0.91)	0.26 0.27	0.99	0.13	0.57
C_24:0_	0.15	0.24 (0.13)	0.19 (0.12)	0.16 (0.24)	0.04 0.03	0.81	0.16	0.43
Branched chain FA (BCFA)								
iso C_13:0_	0.10	0.13 (0.11)	0.06 (0.08)	0.10 (0.11)	0.02 0.01	0.29	0.55	<0.01
iso C_15:0_	0.14	0.36 (0.19)	0.05 (0.06)	0.06 (0.15)	0.05 0.04	0.03	0.07	<0.01
iso C_17:0_	0.51	0.50 (0.54)	0.51 (0.48)	0.46 (0.48)	0.04 0.02	0.44	0.54	0.65
anteiso C_13:0_	0.12	0.12 (0.13)	0.11 (0.12)	0.10 (0.11)	0.02 0.02	0.32	0.84	0.98
anteiso C_15:0_	0.22	0.24 (0.23)	0.18 (0.21)	0.22 (0.20)	0.02 0.01	0.57	0.47	0.05
anteiso C_17:0_	0.65	0.90 (0.73)	1.05 (0.58)	0.55 (0.60)	0.10 0.05	0.70	<0.001	0.19
∑BCFA ^2^	1.95	2.54 (2.27)	2.22 (1.73)	1.71 (1.87)	0.14 0.07	0.08	<0.001	0.26
∑OBCFA ^3^	4.15	4.92 (4.51)	4.16 (3.94)	3.94 (3.93)	0.22 0.19	0.11	0.02	0.03
∑SFA ^4^	66.7	65.4 (66.1)	60.3 (67.1)	69.5 (66.9)	1.3 1.3	0.52	<0.001	0.01
Monounsaturated FA (MUFA)								
C_18:1_ trans-9	0.41	0.44 (0.45)	0.45 (0.37)	0.37 (0.39)	0.04 0.04	0.55	0.15	0.71
C_18:1_ cis-9 (oleic acid)	18.1	19.3 (18.3)	23.5 (18.3)	17.5 (17.9)	1.4 1.2	0.63	<0.01	0.04
C_18:1_ trans-11 (vaccenic acid)	0.97	1.05 (0.98)	1.12 (0.93)	0.90 (1.02)	0.08 0.08	0.74	0.08	0.49
C_24:1_	0.27	0.60 (0.24)	1.01 (0.29)	0.13 (0.25)	0.21 0.08	0.99	<0.01	0.17
∑MUFA ^5^	25.1	26.9 (25.3)	31.2 (25.1)	24.0 (25.3)	1.2 1.0	0.85	<0.001	0.01
Polyunsaturated FA (PUFA)								
C_18:2_ trans-9,12	0.38	0.36 (0.54)	0.28 (0.21)	0.34 (0.36)	0.06 0.06	0.42	0.51	0.52
C_18:2_ cis-9,12 (linoleic acid)	2.48	2.38 (2.92)	2.35 (2.09)	2.25 (2.36)	0.19 0.20	0.38	1.00	0.89
C_18:3_ cis-9 (α-linolenic acid)	0.32	0.34 (0.35)	0.33 (0.30)	0.27 (0.31)	0.03 0.03	0.24	0.27	0.88
C_20:3_	0.15	0.28 (0.11)	0.16 (0.18)	0.11 (0.15)	0.05 0.05	0.27	0.06	0.20
C_22:2_ (Docosadienoic acid)	0.07	0.17 (0.08)	0.19 (0.06)	0.13 (0.08)	0.02 0.02	0.06	<0.01	0.98
C_20:5_ (Eicosapentaenoic acid, EPA)	0.11	0.30 (0.09)	0.52 (0.12)	0.08 (0.14)	0.14 0.03	0.86	0.03	0.29
C_22:6_ (Docosahexaenoic acid, DHA)	0.28	0.47 (0.28)	0.60 (0.28)	0.18 (0.28)	0.11 0.06	0.69	<0.01	0.33
∑n-3 PUFA ^6^	0.87	1.38 (0.84)	1.57 (0.88)	0.64 (0.88)	025 0.12	0.65	<0.01	0.50
∑n-6 PUFA ^7^	3.02	3.01 (3.63)	2.94 (2.45)	2.82 (2.95)	0.23 0.21	0.49	0.81	0.97
n-6/n-3 ratio ^8^	3.81	2.68 (4.60)	3.14 (3.23)	4.15 (3.48)	0.49 0.45	0.50	0.04	0.66
∑PUFA ^9^	3.83	4.38 (4.47)	4.51 (3.33)	3.47 (3.83)	0.35 0.25	0.53	0.02	0.65
∑Unsaturated FA (UFA) ^10^	29.1	31.2 (29.7)	35.9 (28.4)	27.5 (29.2)	1.2 1.0	0.98	<0.001	0.01
∑Other FA	6.69	7.51 (6.73)	7.00 (6.31)	6.06 (7.03)	0.47 0.29	0.24	<0.07	0.68
Atherogenicity index (AI) ^11^	2.91	2.51 (2.87)	1.77 (2.95)	3.12 (2.92)	0.17 0.18	0.87	<0.001	<0.01
h/H ratio ^12^	0.41	0.47 (0.42)	0.70 (0.40)	0.38 (0.40)	0.05 0.03	0.50	<0.001	<0.01
Spreadability index (SI) ^13^	2.46	2.06 (2.35)	1.55 (2.55)	2.43 (2.45)	0.18 0.24	0.44	<0.01	0.09

^1^* FAs = fatty acids; * SE = standard error; ^1^ Values in parentheses are LSM for cows in control group without supplementing amla fruit for each corresponding amla fruit dose; ^2^ ΣBCFA = sum of all branched iso and anteiso FA; ^3^ ΣOBCFA = sum of the odd- and branched-chain fatty acids (11:0, iso 13:0, anteiso 13:0, 13:0, iso 14:0, iso 15:0, anteiso 15:0, 15:0, iso 16:0, iso 17:0, anteiso 17:0, 17:0); ^4^ ΣSFA = C_10:0_ + C_12:0_ + C_13:0_ + C_14:0_ + C_15:0_ + C_16:0_ + C_17:0_ + C_18:0_ + C_19:0_ + C_20:0_ + C_22:0_ + C_24:0_ + C_26:0_; ^5^ ΣMUFA = C_14:1_ + C_16:1_ + C_18:1_ + C_20:1_; ^6^ Σn-3 PUFA = C_18:3_ n-3 + C_20:5_ n-3 + C_22:6_ n-3; ^7^ Σn-6 PUFA = C_18:2_ n-6 + C_18:3_ n-6 + C_20:2_ n-6 + C_20:3_ n-6 + C_20:4_ n-6 + C_22:4_ n; ^8^ ratio between n-6 PUFA and n-3 PUFA; ^9^ ΣPUFA = total n-3 PUFA + total n-6 PUFA; ^10^ ΣUFA = ∑MUFA + ∑PUFA; ^11^ the atherogenicity index (AI) defined as [(C_12:0_ + 4 * C_14:0_ + C_16:0_)/(ƩUFA)]; ^12^ The hypocholesterolemic and hypercholesterolemic (h/H) fatty acids ratio (h:H) = (cis s C_18:1_ + ΣPUFA)/(C_12:0_ + C_14:0_ + C_16:0_); ^13^ SI = calculated as C_16:0_/C_18:1_ cis-9.

**Table 5:** Eigen analysis and correlations between variables and PC.

	PC1	PC2	PC3	PC4
C_10:0_	−0.502	0.317	−0.190	0.293
C_12:0_	−0.680	0.349	−0.214	0.119
*iso* C_13:0_	−0.301	0.429	−0.557	0.285
C_14:0_	−0.807	0.295	−0.027	−0.120
*iso* C_15:0_	−0.061	0.392	−0.795	0.132
C_15:0_	−0.630	0.369	0.120	−0.574
C_16:0_ (palmitic acid)	−0.909	0.133	0.096	−0.092
*anteiso* C_17:0_	0.751	0.501	0.144	−0.155
C_17:0_	0.242	0.004	−0.037	−0.854
C_18:0_ (stearic acid)	0.634	−0.574	−0.046	−0.176
C_18:1_ trans-11 (vaccenic acid)	0.182	−0.425	−0.474	−0.180
C1_8:1_ cis-9 (oleic acid)	0.540	−0.766	−0.256	−0.104
C_20:0_	0.755	0.452	0.325	0.085
C_21:0_	0.658	0.410	0.423	0.147
C_22:0_	0.727	0.426	0.370	0.087
C_22:2_ (Docosadienoic acid)	0.542	0.088	0.026	0.264
C_20:5_ (Eicosapentaenoic acid, EPA)	0.716	0.440	0.121	0.011
C_22:6_ (Docosahexaenoic acid, DHA)	0.775	0.517	0.016	0.094
∑BCFA ^1^	0.575	0.648	−0.420	−0.096
∑OBCFA ^2^	0.144	0.750	−0.331	−0.530
∑SFA ^3^	−0.870	0.247	0.166	−0.213
∑MUFA ^4^	0.700	−0.638	−0.247	−0.096
∑PUFA ^5^	0.681	0.509	−0.115	0.020
∑UFA ^6^	0.816	−0.466	−0.258	−0.084
∑isoFA	0.049	0.401	−0.857	0.066
∑antisoFA	0.734	0.567	0.078	−0.178
∑PUFAn-3 ^7^	0.777	0.512	0.086	0.059
Atherogenicity index (AI) ^8^	−0.911	0.335	0.144	−0.078
h/H ratio ^9^	0.840	−0.452	−0.145	−0.077
Eigenvalue	12.381	6.159	3.043	1.898
Variability (%)	42.694	21.237	10.492	6.544
Cumulative %	42.694	63.931	74.423	80.967

^1^ ΣBCFA = sum of all branched iso and anteiso FA; ^2^ ΣOBCFA = sum of the odd- and branched-chain fatty acids (11:0, *iso* 13:0, *anteiso* 13:0, 13:0, *iso* 14:0, *iso* 15:0, *anteiso* 15:0, 15:0, *iso* 16:0, *iso* 17:0, *anteiso* 17:0, 17:0); ^3^ ΣSFA = C_10:0_ + C_12:0_ + C_13:0_ + C_14:0_ + C_15:0_ + C_16:0_ + C_17:0_ + C_18:0_ + C_19:0_ + C_20:0_ + C_22:0_ + C_24:0_ + C_26:0_; ^4^ Σ MUFA = C_14:1_ + C_16:1_ + C_18:1_ + C_20:1_; ^5^ ΣPUFA = C_18:3n-3_ + C_20:3n-3_ + C_20:5n-3_ + C_22:6n-3_ + C_18:2n-6_ + C_18:3n-6_ + C_20:2n-6_ + C_20:3n-6_ + C_20:4n-6_ + C_22:4n_; ^6^ ΣUFA = ∑MUFA + ∑PUFA; ^7^ Σn-3 PUFA = C_18:3n-3_ + C_20:3n-3_ + C_20:5n-3_ + C_22:6n-3_; ^8^ the atherogenicity index (AI) defined as [(C_12:0_ + 4 ∗ C_14:0_ + C_16:0_)/(Ʃ UFA)]; ^9^ the hypocholesterolemic and hypercholesterolemic (h/H) fatty acids ratio (h:H) = (cis s C_18:1_ + ΣPUFA)/(C_12:0_ + C_14:0_ + C_16:0_).

**Table 6:** Serum and plasma biochemistry of lactating cows fed a TMR without supplementation (control, 0 g/d), or supplemented with 200, 400 or 600 g/d amla fruit doses.

Item	Fresh Amla Fruit Doses Supplemented g/Day					Treatment by Dose, *p*		
	0	200	400	600	SE	Linear	Quadratic	Cubic
Serum blood chemistry								
β-hydroxybutyrate	0.22	0.27 (0.18)	0.25 (0.26)	0.29 (0.23)	0.03 0.04	0.26	0.89	0.35
Glucose	70.2	85.6 (67.0)	84.5 (71.4)	76.5 (72.0)	4.1 3.8	0.27	<0.01	0.61
Non-esterified fatty acid	0.30	0.31 (0.37)	0.23 (0.30)	0.33 (0.23)	0.04 0.05	0.88	0.28	0.23
Triglycerides	0.12	0.12 (0.11)	0.10 (0.13)	0.11 (0.13)	0.02 0.02	0.44	0.68	0.36
Total cholesterol	5.17	5.47 (5.00)	5.95 (5.36)	4.51 (5.16)	0.34 0.42	0.26	0.01	0.19
Total protein	55.3	58.2 (53.1)	65.9 (56.7)	43.8 (56.3)	2.3 2.5	0.01	<0.001	0.01
Albumin (A)	22.8	20.5 (21.2)	31.4 (24.1)	21.8 (23.2)	1.2 1.5	0.14	0.01	<0.001
Globulin (G)	32.5	37.8 (31.9)	34.4 (32.5)	19.4 (33.1)	2.3 2.4	<0.001	<0.001	0.78
A/G ratio	0.74	0.56 (0.68)	1.06 (0.79)	1.21 (0.74)	0.10 0.17	<0.001	0.13	0.06
Aspartate aminotransferase	10.7	9.38 (11.0)	9.79 (10.8)	9.79 (10.3)	0.44 0.50	0.23	0.15	0.31
Gamma-Glutamyl Transferase	13.7	16.2 (11.0)	16.0 (15.2)	17.1 (14.7)	1.5 1.6	0.12	0.65	0.57
Alanine aminotransferase	11.0	14.8 (10.5)	11.4 (11.0)	12.0 (11.5)	1.4 1.8	0.94	0.28	0.11
Plasma blood chemistry								
Creatine	59.4	60.0 (58.8)	59.1 (59.0)	59.9 (61.0)	2.9 2.5	0.96	0.98	0.83
Lactic acid	0.93	1.10 (1.01)	0.85 (0.79)	0.90 (0.98)	0.07 0.07	0.30	0.41	0.04
Uric acid	47.0	50.9 (46.1)	50.1 (46.9)	50.7 (49.2)	2.3 4.7	0.27	0.48	0.60

**Table 7:** Plasma and milk antioxidant capacity of lactating cows (n = 8) fed a TMR without supplementation (control, 0 g/d), or supplemented with 200, 400 or 600 g/d amla fruit doses.

Item	Fresh Amla Fruit Doses Supplemented g/Day					Treatment by Dose, *p*		
	0	200	400	600	SE	Linear	Quadratic	Cubic
Plasma								
Malondialdehyde (MDA)	12.6	12.1 (14.0)	12.8 (12.5)	11.6 (13.7)	1.1 0.97	0.61	0.76	0.57
Nitric oxide (NO)	10.5	10.8(9.55)	10.5(11.8)	6.07(10.1)	1.1 1.2	<0.01	0.04	0.51
Superoxide dismutase (SOD)	22.4	20.5(22.2)	25.2(22.1)	24.1(22.7)	0.92 0.78	0.01	0.63	<0.01
Milk								
Total antioxidant capacity (TAC)	0.97	1.00 (0.95)	1.09 (1.02)	1.09 (1.05)	0.05 0.06	<0.01	0.65	0.32
ferric reducing-antioxidant power (FRAP)	0.62	0.49 (0.53)	0.75 (0.65)	0.57 (0.68)	0.03 0.04	0.43	0.45	<0.001
Flavonoid	42.9	63.0 (44.5)	66.7 (48.7)	36.7 (35.5)	3.1 3.5	0.32	<0.001	0.32
Ascorbic	0.03	0.04 (0.03)	0.06 (0.03)	0.03 (0.03)	0.01 0.01	0.49	0.01	0.15

## Data Availability

Data is contained within the article and supplementary material.

## Supplementary Materials

The following supporting information can be downloaded at: https://www.mdpi.com/article/10.3390/antiox11030485/s1, Table S1: Classification of the 514 detected metabolites in fresh amla fruit; Table S2: A list of the 514 metabolites detected in fresh amla (Phyllanthus emblica ) fruit; Table S3: Table S3 Top 15 compounds identified by UPLC–MS/MS method operating in ESI mode in Fresh amla fruit; Table S4: Pairwise correlation coefficients between individual FA1 profiles (g/100 g of total fatty acids) or groupings of fatty acids [37].
