# Supplementary material for: Fresh Phyllanthus emblica (Amla) Fruit Supplementation Enhances Milk Fatty Acid Profiles and the Antioxidant Capacities of Milk and Blood in Dairy Cows
Source: Antioxidants (Basel). 2022 Feb 28;11(3):485. doi: 10.3390/antiox11030485 (PMC8944803; doi:10.3390/antiox11030485)
Supplement: Supplementary file 1 [file antioxidants-11-00485-s001.zip › Table S1.pdf]

Table S1 Classification of the 514 detected metabolites in fresh amla fruit

Primary classification	Secondary classification	Number of metabolites
Phenolic acids	Phenolic acids	113
Lipids	Free fatty acids	49
	Glycerol ester	12
	Lysophosphatidylcholine	22
	Lysophosphatidylethanolamine	17
	Phosphatidylcholines	1
	Sphingolipids	1
Flavonoids	Anthocyanidins	1
	Chalcones	4
	Flavanols	11
	Flavanones	15
	Flavanonols	6
	Flavones	21
	Flavonoid carbonoside	2
	Flavonols	38
	Isoflavones	3
Amino acids and derivatives	Amino acids and derivatives	46
Tannins	Tannin	34
	Proanthocyanidins	4
Alkaloids	Alkaloids	10
	Phenolamine	1
	Plumerane	4
	Pyridine alkaloids	1
	Pyrrole alkaloids	2
	Piperidine alkaloids	1
Organic acids	Organic acids	16
Terpenoids	Ditepenoids	1
	Monoterpenoids	4
	Sesquiterpenoids	2
	Triterpene	9
Lignans and Coumarins	Coumarins	2
	Lignans	10
Nucleotides and derivatives	Nucleotides and derivatives	10
Others	Saccharides and Alcohols	24
	Stilbene	1
	Vitamin	8
	Others	8
