# Supplementary material for: Fresh Phyllanthus emblica (Amla) Fruit Supplementation Enhances Milk Fatty Acid Profiles and the Antioxidant Capacities of Milk and Blood in Dairy Cows
Source: Antioxidants (Basel). 2022 Feb 28;11(3):485. doi: 10.3390/antiox11030485 (PMC8944803; doi:10.3390/antiox11030485)
Supplement: Supplementary file 1 [file antioxidants-11-00485-s001.zip › Table S3.pdf]

Table S3 Top 15 compounds identified by UPLC–MS/MS method operating in ESI mode in Fresh amla fruit

No	Index	R_T (min)	Identified compound	Calibration diagrams
1	lmyn001519	2.02	L-Malic acid-2-O-gallate	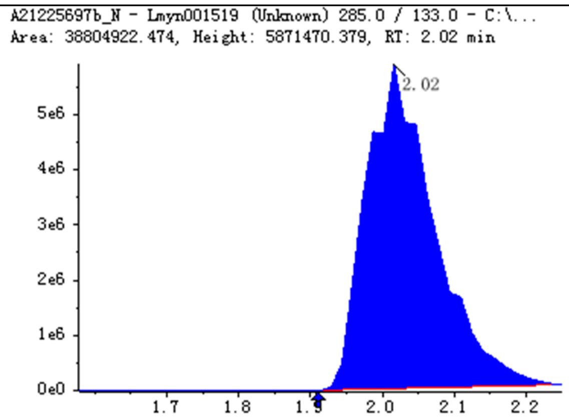
2	pmp001309	3.82	6-Hydroxykaempferol-7-O-glucoside	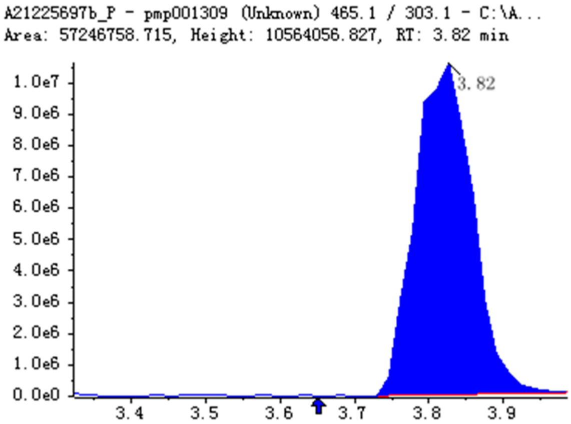
3	Pmn001513	2.88	Digallic Acid	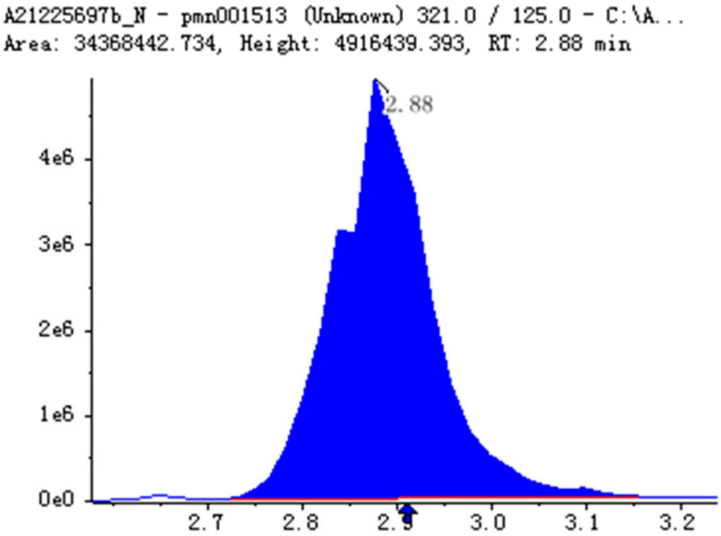

4	Lmyn002788	3.13	Methyl gallate	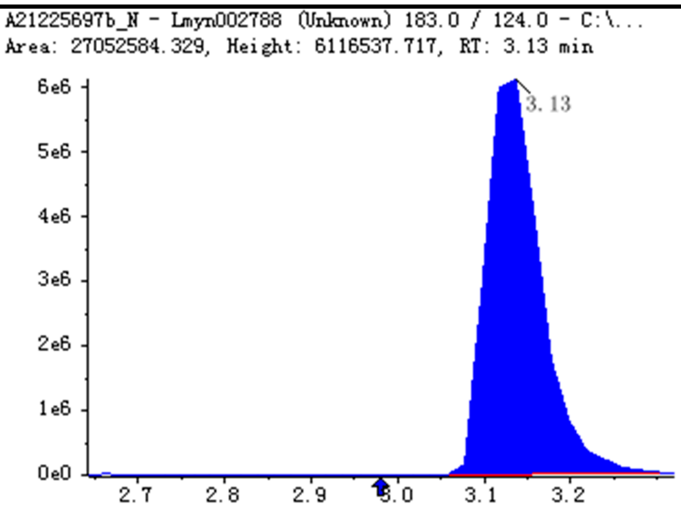
5	Xmbp001692	1.44	2,4,6-Trihy-droxybenzoic acid	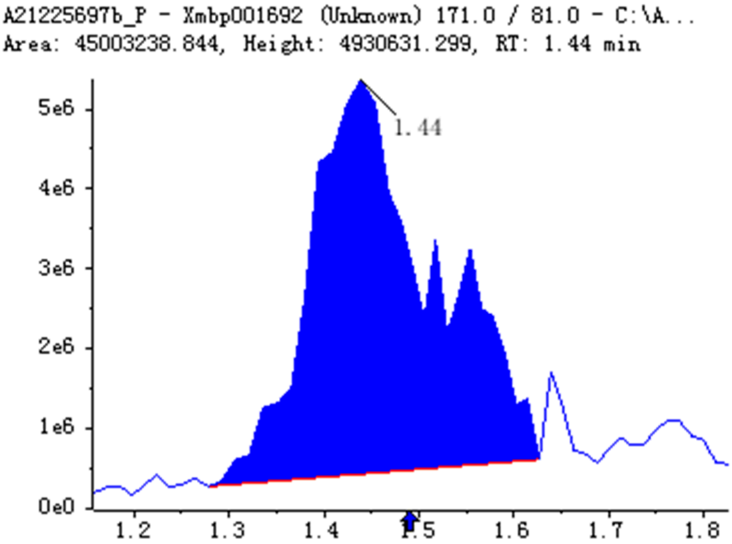
6	Zmhn005413	4.09	p-Dimeric galloyl methyl ester	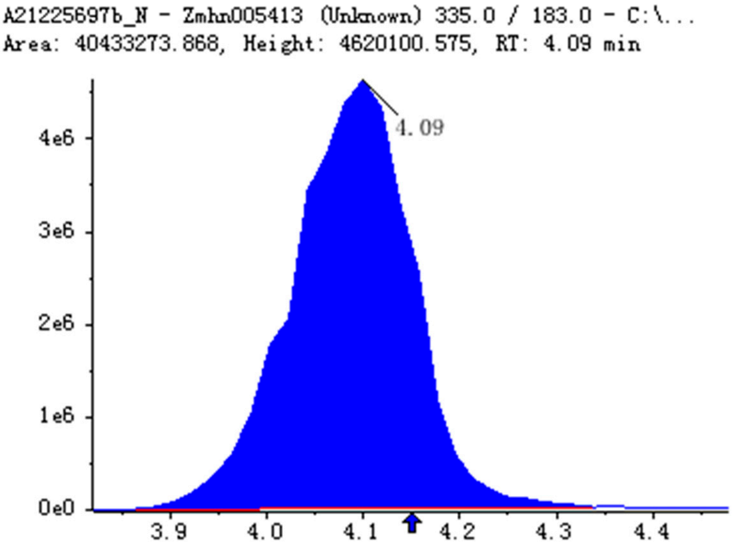

7	Wmhn001495	2.70	1,4-Di-O-Galloyl-D-glucose	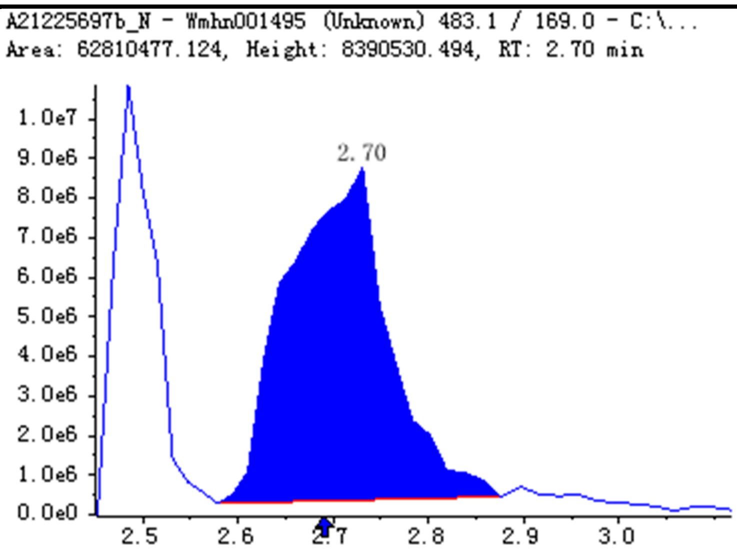
8	Pme2246	3.81	Ellagic acid	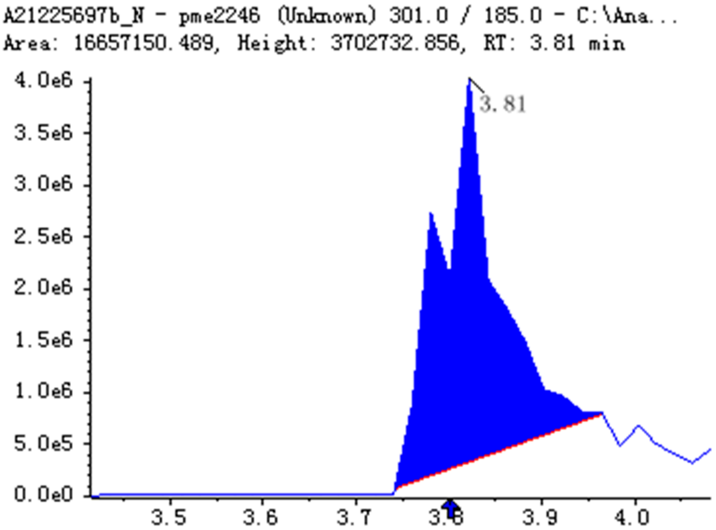
9	MWSmce341	9.66	Butyl isobutyl phthalate*	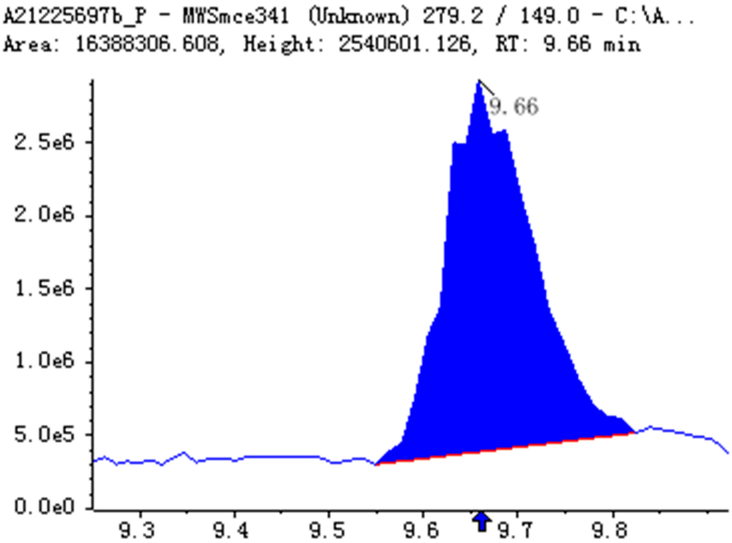

10	pmn001516	2.28	5-Galloylshikimic acid	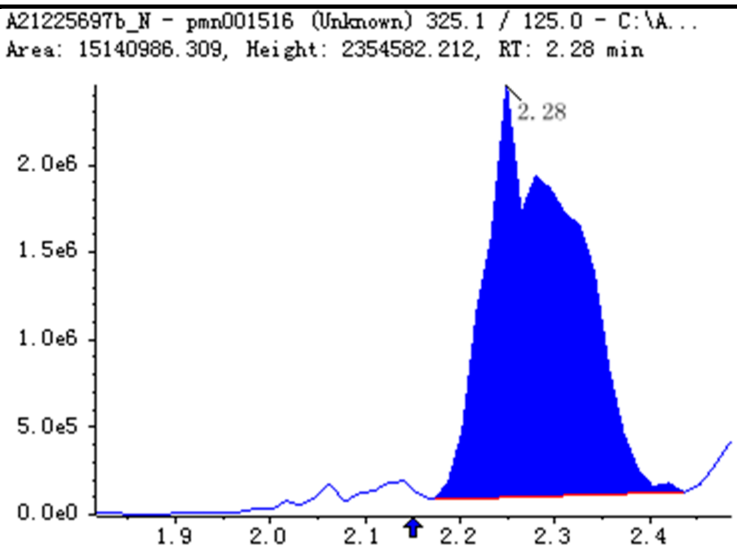
11	pmn001518	2.15	1-O-Galloyl-D-glucose	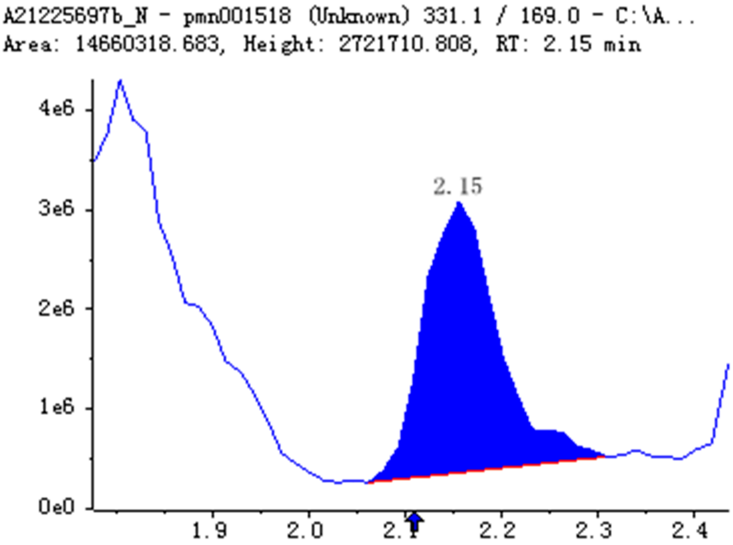
12	mws0056	3.76	Myricetin-3-O-rhamnoside (Myricitrin)	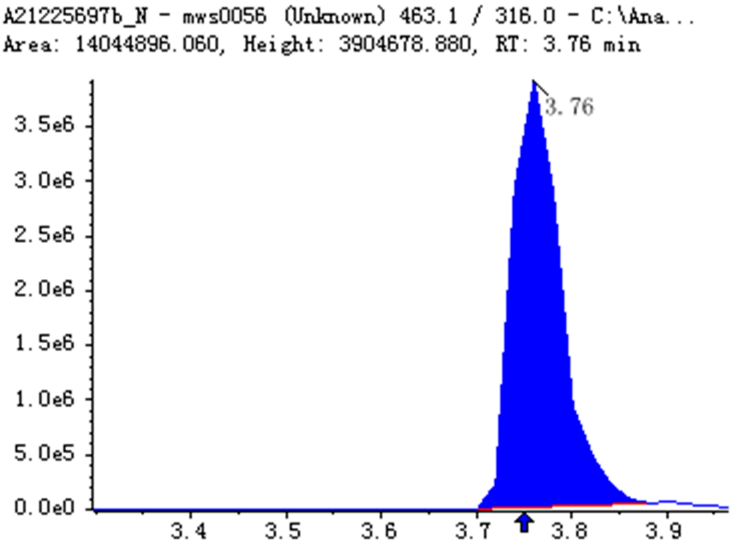

13	mws0355	3.86	Catechin gallate*	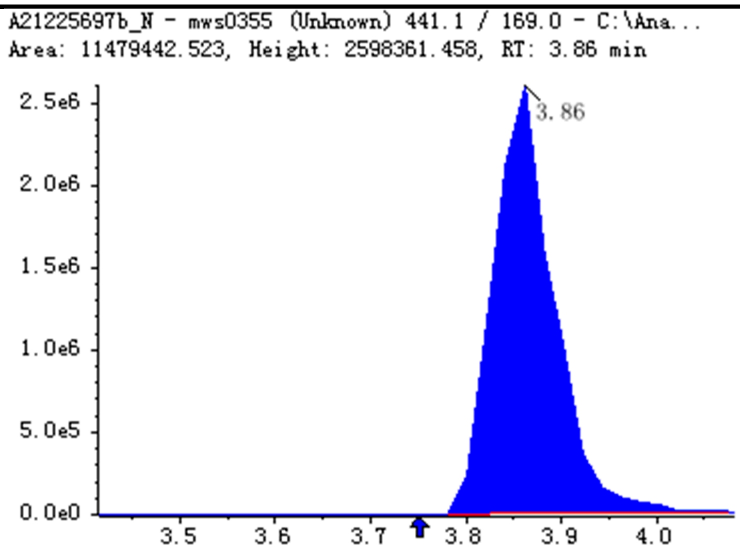
14	mws0061	3.82	Quercetin-3-O-galactoside (Hyperin)	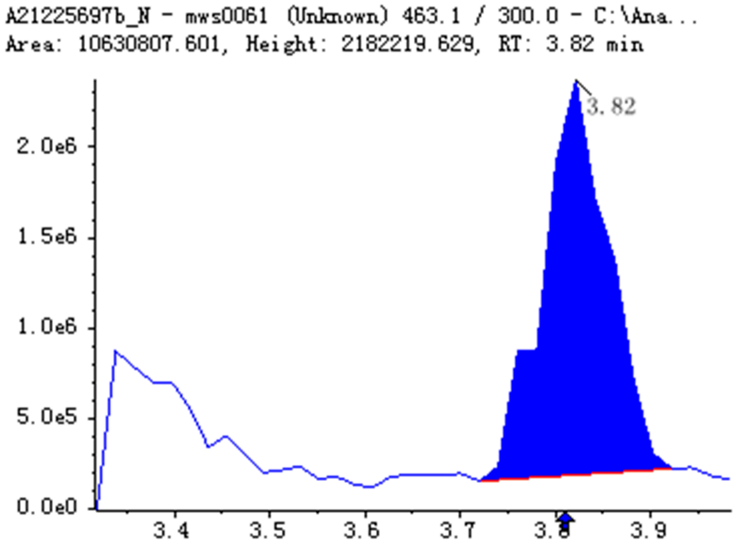
15	Lmyn002530	2.55	Carpinusin	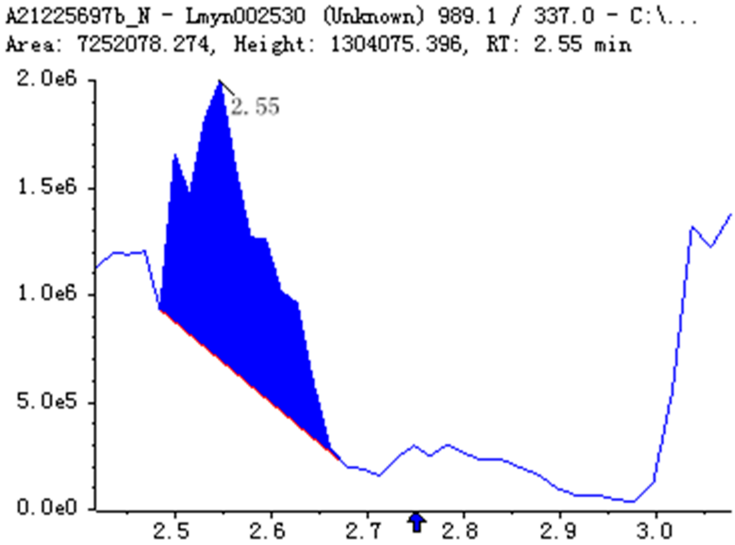
