# Supplementary material for: Fresh Phyllanthus emblica (Amla) Fruit Supplementation Enhances Milk Fatty Acid Profiles and the Antioxidant Capacities of Milk and Blood in Dairy Cows
Source: Antioxidants (Basel). 2022 Feb 28;11(3):485. doi: 10.3390/antiox11030485 (PMC8944803; doi:10.3390/antiox11030485)
Supplement: Supplementary file 1 [file antioxidants-11-00485-s001.zip › Table S4.pdf]

Table S4:Pairwise correlation coefficients between individual FA[1] profiles (g/100 g of total fatty acids) or groupings of fatty acids

Variable	by Variable	Correlation	Lower 95%	Upper 95%	Signif Prob
C14:0	C12:0	0.8297	0.7070	0.9039	<.0001*
C16:0	C12:0	0.4767	0.2094	0.6777	0.0011*
C16:0	C14:0	0.6570	0.4474	0.7982	<.0001*
C18:0	C12:0	-0.5601	-0.7348	-0.3157	<.0001*
C18:0	C14:0	-0.6058	-0.7651	-0.3767	<.0001*
C18:0	C16:0	-0.7107	-0.8320	-0.5245	<.0001*
C20:5	C12:0	-0.3179	-0.5618	-0.0232	0.0355*
C20:5	C14:0	-0.4283	-0.6434	-0.1506	0.0037*
C20:5	C16:0	-0.5351	-0.7180	-0.2833	0.0002*
C20:5	C18:0	0.1519	-0.1518	0.4294	0.3249
C22:6	C12:0	-0.3745	-0.6042	-0.0873	0.0123*
C22:6	C14:0	-0.5017	-0.6950	-0.2406	0.0005*
C22:6	C16:0	-0.6034	-0.7635	-0.3734	<.0001*
C22:6	C18:0	0.1766	-0.1269	0.4499	0.2514
C22:6	C20:5	0.8076	0.6718	0.8909	<.0001*
Denovo FA	C12:0	0.9394	0.8911	0.9667	<.0001*
Denovo FA	C14:0	0.9480	0.9062	0.9715	<.0001*
Denovo FA	C16:0	0.6222	0.3990	0.7758	<.0001*
Denovo FA	C18:0	-0.6613	-0.8009	-0.4534	<.0001*
Denovo FA	C20:5	-0.3829	-0.6104	-0.0970	0.0103*
Denovo FA	C22:6	-0.4419	-0.6531	-0.1669	0.0027*
Preformed FA	C12:0	-0.6589	-0.7994	-0.4500	<.0001*
Preformed FA	C14:0	-0.7646	-0.8651	-0.6051	<.0001*
Preformed FA	C16:0	-0.9060	-0.9479	-0.8333	<.0001*
Preformed FA	C18:0	0.8943	0.8133	0.9413	<.0001*
Preformed FA	C20:5	0.4356	0.1593	0.6486	0.0031*
Preformed FA	C22:6	0.4473	0.1735	0.6570	0.0023*
Preformed FA	Denovo FA	-0.7794	-0.8741	-0.6278	<.0001*
Mixed FA	C12:0	0.4619	0.1912	0.6673	0.0016*
Mixed FA	C14:0	0.6442	0.4295	0.7900	<.0001*
Mixed FA	C16:0	0.9969	0.9942	0.9983	<.0001*
Mixed FA	C18:0	-0.7113	-0.8324	-0.5253	<.0001*
Mixed FA	C20:5	-0.5316	-0.7156	-0.2788	0.0002*
Mixed FA	C22:6	-0.6080	-0.7665	-0.3797	<.0001*
Mixed FA	Denovo FA	0.6105	0.3831	0.7682	<.0001*
Mixed FA	Preformed FA	-0.9020	-0.9457	-0.8264	<.0001*
OCFA[2]	C12:0	0.3430	0.0513	0.5807	0.0227*
OCFA	C14:0	0.5451	0.2962	0.7247	0.0001*
OCFA	C16:0	0.5121	0.2538	0.7022	0.0004*
OCFA	C18:0	-0.3983	-0.6216	-0.1150	0.0074*
OCFA	C20:5	-0.1582	-0.4346	0.1455	0.3052
OCFA	C22:6	-0.1674	-0.4423	0.1363	0.2775
OCFA	Denovo FA	0.4881	0.2236	0.6856	0.0008*
OCFA	Preformed FA	-0.4695	-0.6726	-0.2005	0.0013*
OCFA	Mixed FA	0.4928	0.2295	0.6889	0.0007*
BCFA[3]	C12:0	-0.1026	-0.3877	0.2004	0.5076
BCFA	C14:0	-0.2498	-0.5089	0.0509	0.1020
BCFA	C16:0	-0.4707	-0.6735	-0.2021	0.0013*
BCFA	C18:0	0.0362	-0.2635	0.3295	0.8156

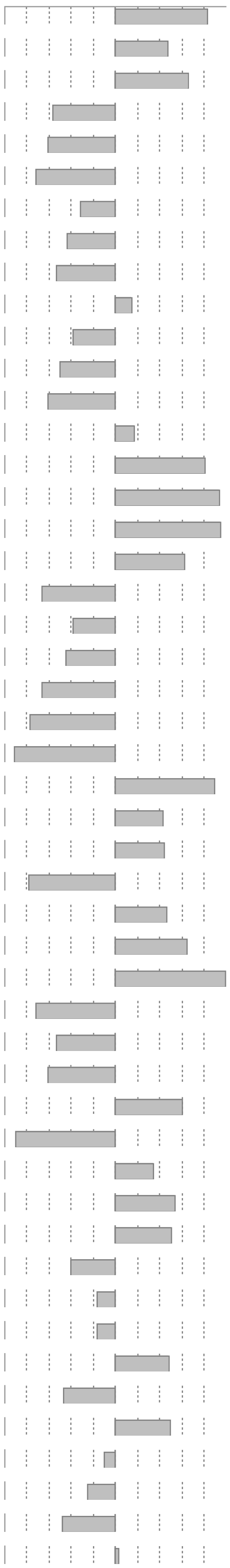

Variable	by Variable	Correlation	Lower 95%	Upper 95%	Signif Prob
BCFA	C20:5	0.5991	0.3676	0.7607	<.0001*
BCFA	C22:6	0.7379	0.5647	0.8488	<.0001*
BCFA	Denovo FA	-0.1590	-0.4354	0.1447	0.3025
BCFA	Preformed FA	0.2760	-0.0227	0.5295	0.0697
BCFA	Mixed FA	-0.4736	-0.6755	-0.2056	0.0012*
BCFA	OCFA	0.0513	-0.2493	0.3430	0.7407
OBCFA [4]	C12:0	0.1421	-0.1616	0.4212	0.3577
OBCFA	C14:0	0.1616	-0.1421	0.4375	0.2947
OBCFA	C16:0	-0.0230	-0.3177	0.2758	0.8824
OBCFA	C18:0	-0.2263	-0.4903	0.0756	0.1396
OBCFA	C20:5	0.3429	0.0512	0.5807	0.0227*
OBCFA	C22:6	0.4398	0.1644	0.6516	0.0028*
OBCFA	Denovo FA	0.1925	-0.1107	0.4629	0.2107
OBCFA	Preformed FA	-0.0941	-0.3803	0.2086	0.5436
OBCFA	Mixed FA	-0.0373	-0.3305	0.2625	0.8101
OBCFA	OCFA	0.6735	0.4708	0.8087	<.0001*
OBCFA	BCFA	0.7728	0.6176	0.8700	<.0001*
SFA [5]	C12:0	0.6289	0.4083	0.7801	<.0001*
SFA	C14:0	0.8158	0.6848	0.8958	<.0001*
SFA	C16:0	0.8616	0.7589	0.9225	<.0001*
SFA	C18:0	-0.5443	-0.7242	-0.2951	0.0001*
SFA	C20:5	-0.4748	-0.6764	-0.2072	0.0011*
SFA	C22:6	-0.5750	-0.7448	-0.3354	<.0001*
SFA	Denovo FA	0.7659	0.6071	0.8659	<.0001*
SFA	Preformed FA	-0.7997	-0.8862	-0.6593	<.0001*
SFA	Mixed FA	0.8482	0.7369	0.9148	<.0001*
SFA	OCFA	0.6465	0.4327	0.7915	<.0001*
SFA	BCFA	-0.4051	-0.6266	-0.1230	0.0064*
SFA	OBCFA	0.1111	-0.1921	0.3949	0.4729
MUFA [6]	C12:0	-0.6299	-0.7808	-0.4096	<.0001*
MUFA	C14:0	-0.7132	-0.8336	-0.5281	<.0001*
MUFA	C16:0	-0.7380	-0.8489	-0.5649	<.0001*
MUFA	C18:0	0.8253	0.6999	0.9013	<.0001*
MUFA	C20:5	0.2201	-0.0822	0.4853	0.1511
MUFA	C22:6	0.2062	-0.0966	0.4741	0.1792
MUFA	Denovo FA	-0.7334	-0.8460	-0.5580	<.0001*
MUFA	Preformed FA	0.8976	0.8190	0.9432	<.0001*
MUFA	Mixed FA	-0.7229	-0.8396	-0.5425	<.0001*
MUFA	OCFA	-0.4997	-0.6937	-0.2381	0.0006*
MUFA	BCFA	0.0937	-0.2090	0.3800	0.5452
MUFA	OBCFA	-0.2482	-0.5077	0.0525	0.1042
MUFA	SFA	-0.7880	-0.8792	-0.6410	<.0001*
PUFA [7]	C12:0	-0.1290	-0.4102	0.1745	0.4039
PUFA	C14:0	-0.3165	-0.5607	-0.0216	0.0363*
PUFA	C16:0	-0.6240	-0.7769	-0.4015	<.0001*
PUFA	C18:0	0.1125	-0.1907	0.3962	0.4672
PUFA	C20:5	0.7217	0.5406	0.8388	<.0001*
PUFA	C22:6	0.7980	0.6567	0.8852	<.0001*
PUFA	Denovo FA	-0.2323	-0.4950	0.0694	0.1292
PUFA	Preformed FA	0.4304	0.1531	0.6449	0.0035*

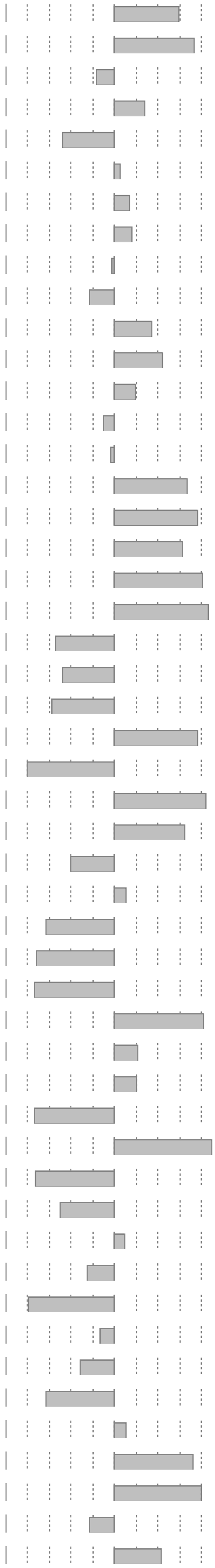

Variable	by Variable	Correlation	Lower 95%	Upper 95%	Signif Prob
PUFA	Mixed FA	-0.6267	-0.7787	-0.4053	<.0001*
PUFA	OCFA	-0.0698	-0.3592	0.2319	0.6527
PUFA	BCFA	0.6970	0.5045	0.8235	<.0001*
PUFA	OBCFA	0.4715	0.2031	0.6740	0.0012*
PUFA	SFA	-0.5223	-0.7092	-0.2668	0.0003*
PUFA	MUFA	0.1772	-0.1263	0.4504	0.2498
UFA[8]	C12:0	-0.6154	-0.7714	-0.3897	<.0001*
UFA	C14:0	-0.7387	-0.8493	-0.5660	<.0001*
UFA	C16:0	-0.8375	-0.9085	-0.7195	<.0001*
UFA	C18:0	0.7924	0.6479	0.8818	<.0001*
UFA	C20:5	0.3817	0.0956	0.6095	0.0106*
UFA	C22:6	0.3876	0.1026	0.6139	0.0093*
UFA	Denovo FA	-0.7367	-0.8481	-0.5629	<.0001*
UFA	Preformed FA	0.9377	0.8880	0.9657	<.0001*
UFA	Mixed FA	-0.8242	-0.9007	-0.6981	<.0001*
UFA	OCFA	-0.4802	-0.6801	-0.2137	0.0010*
UFA	BCFA	0.2585	-0.0416	0.5158	0.0902
UFA	OBCFA	-0.1138	-0.3973	0.1894	0.4619
UFA	SFA	-0.8587	-0.9208	-0.7541	<.0001*
UFA	MUFA	0.9702	0.9457	0.9837	<.0001*
UFA	PUFA	0.4105	0.1294	0.6305	0.0056*
AI[9]	C12:0	0.7085	0.5212	0.8306	<.0001*
AI	C14:0	0.8566	0.7507	0.9196	<.0001*
AI	C16:0	0.9053	0.8320	0.9475	<.0001*
AI	C18:0	-0.7741	-0.8708	-0.6196	<.0001*
AI	C20:5	-0.4611	-0.6667	-0.1903	0.0016*
AI	C22:6	-0.5364	-0.7188	-0.2849	0.0002*
AI	Denovo FA	0.8313	0.7095	0.9049	<.0001*
AI	Preformed FA	-0.9448	-0.9697	-0.9005	<.0001*
AI	Mixed FA	0.8903	0.8066	0.9390	<.0001*
AI	OCFA	0.5837	0.3469	0.7505	<.0001*
AI	BCFA	-0.3587	-0.5925	-0.0692	0.0168*
AI	OBCFA	0.1055	-0.1976	0.3901	0.4956
AI	SFA	0.9156	0.8498	0.9534	<.0001*
AI	MUFA	-0.8821	-0.9343	-0.7929	<.0001*
AI	PUFA	-0.4635	-0.6684	-0.1932	0.0015*
AI	UFA	-0.9315	-0.9623	-0.8772	<.0001*
Hh[10]	C12:0	-0.6541	-0.7963	-0.4433	<.0001*
Hh	C14:0	-0.8010	-0.8870	-0.6614	<.0001*
Hh	C16:0	-0.8490	-0.9152	-0.7382	<.0001*
Hh	C18:0	0.7935	0.6497	0.8825	<.0001*
Hh	C20:5	0.3967	0.1131	0.6205	0.0077*
Hh	C22:6	0.3999	0.1169	0.6229	0.0072*
Hh	Denovo FA	-0.7936	-0.8825	-0.6497	<.0001*
Hh	Preformed FA	0.9438	0.8987	0.9691	<.0001*
Hh	Mixed FA	-0.8435	-0.9120	-0.7292	<.0001*
Hh	OCFA	-0.4875	-0.6852	-0.2228	0.0008*
Hh	BCFA	0.2385	-0.0629	0.5000	0.1190
Hh	OBCFA	-0.1333	-0.4138	0.1703	0.3884
Hh	SFA	-0.8729	-0.9290	-0.7775	<.0001*

Variable	by Variable	Correlation	Lower 95%	Upper 95%	Signif Prob
Hh	MUFA	0.9369	0.8866	0.9653	<.0001*
Hh	PUFA	0.4172	0.1374	0.6354	0.0048*
Hh	UFA	0.9708	0.9468	0.9841	<.0001*
Hh	AI	-0.9173	-0.9543	-0.8527	<.0001*
SI[11]	C12:0	0.4746	0.2069	0.6762	0.0011*
SI	C14:0	0.6041	0.3743	0.7640	<.0001*
SI	C16:0	0.8867	0.8006	0.9370	<.0001*
SI	C18:0	-0.8344	-0.9067	-0.7145	<.0001*
SI	C20:5	-0.2960	-0.5450	0.0010	0.0511
SI	C22:6	-0.3243	-0.5666	-0.0303	0.0317*
SI	Denovo FA	0.6000	0.3688	0.7613	<.0001*
SI	Preformed FA	-0.9165	-0.9539	-0.8513	<.0001*
SI	Mixed FA	0.8782	0.7864	0.9321	<.0001*
SI	OCFA	0.5453	0.2965	0.7249	0.0001*
SI	BCFA	-0.2058	-0.4737	0.0970	0.1801
SI	OBCFA	0.1943	-0.1089	0.4644	0.2064
SI	SFA	0.7807	0.6297	0.8748	<.0001*
SI	MUFA	-0.8887	-0.9381	-0.8040	<.0001*
SI	PUFA	-0.3149	-0.5595	-0.0199	0.0373*
SI	UFA	-0.9010	-0.9451	-0.8247	<.0001*
SI	AI	0.9035	0.8291	0.9465	<.0001*
SI	Hh	-0.8557	-0.9191	-0.7491	<.0001*

[1] * FAs= fatty acids; [2] OCFA= odd chain FA; [3] BCFA=Branched chain FA ;[4] OBCFA=Sum of the odd- and branched-chain fatty acids;[5] SFA= total sum of saturated FA; [6] MUFA =total sum of Mono unsaturated FA; [7] PUFA = total sum of Poly unsaturated FA;[8] UFA = total sum of unsaturated FA; [9] AI= the atherogenicity index; [10] h/H= The hypocholesterolemic and hypercholesterolemic fatty acids ratio (h:H); [11] SI=spreadability index calculated as 16:0/18:1 cis-9 Drackley *et al.* [1]

1. Drackley, J.K.; Overton, T.R.; Ortiz-Gonzalez, G.; Beaulieu, A.D.; Barbano, D.M.; Lynch, J.M.; Perkins, E.G. Responses to increasing amounts of high-oleic sunflower fatty acids infused into the abomasum of lactating dairy cows. *J. Dairy Sci.* **2007**, *90*, 5165-5175, doi:10.3168/jds.2007-0122.
